# Revisiting Querleu–Morrow Radical Hysterectomy: How to Apply the Anatomy of Parametrium and Pelvic Autonomic Nerves to Cervical Cancer Surgery?

**DOI:** 10.3390/cancers16152729

**Published:** 2024-07-31

**Authors:** Stoyan Kostov, Yavor Kornovski, Rafał Watrowski, Angel Yordanov, Stanislav Slavchev, Yonka Ivanova, Hakan Yalcin, Ivan Ivanov, Ilker Selcuk

**Affiliations:** 1 Research Institute, Medical University Pleven, 5800 Pleven, Bulgaria; drstoqn.kostov@gmail.com; 2 Department of Gynecology, Hospital “Saint Anna”, Medical University “Prof. Dr. Paraskev Stoyanov”, 9002 Varna, Bulgaria; ykornovski@abv.bg (Y.K.); st_slavchev@abv.bg (S.S.); yonka.ivanova@abv.bg (Y.I.); 3 Department of Obstetrics and Gynecology, Helios Hospital Müllheim, 79379 Müllheim, Germany; rafal.watrowski@gmx.at; 4 Faculty Associate, Medical Center, University of Freiburg, 79106 Freiburg, Germany; 5 Department of Gynecologic Oncology, Medical University Pleven, 5800 Pleven, Bulgaria; 6 Department of Gynecologic Oncology, Ankara Bilkent City Hospital, Maternity Hospital, 06800 Ankara, Turkey; drhyalcin@yahoo.com (H.Y.); ilkerselcukmd@hotmail.com (I.S.); 7 Department of General and Clinical Pathology, University Hospital “Dr. Georgi Stranski”, 5800 Pleven, Bulgaria; posledenzalez@gmail.com

**Keywords:** radical hysterectomy, Querleu and Morrow classification, cervical cancer surgery, parametrium, nerve-sparing hysterectomy, surgical anatomy

## Abstract

**Simple Summary:**

Radical hysterectomy, mainly used for the treatment of cancer of the uterine cervix, was introduced over a century ago. Since then, various and many different classifications of radical hysterectomy, which diverge in terms of terminology, radicality, or anatomical landmarks, have been described. The recently introduced Querleu and Morrow (Q–M) classification of radical hysterectomy provides a unique opportunity for uniform surgical and anatomical terminology. The Q–M classification provides precise explanations of anatomical landmarks and resection margins for the three uterus parametria. However, there are still some disagreements and misconceptions regarding the terminology and anatomical landmarks of the Q–M classification. Therefore, we highlight the surgical anatomy of the Q–M radical hysterectomy classification and define the importance of anatomical landmarks. We proposed an update of the Q–M classification that may facilitate surgical harmonization and precise standardization among oncogynecologists, which could yield accurate and comparable results from multicenter surgical clinical studies.

**Abstract:**

In 2008, Querleu and Morrow proposed a novel classification of radical hysterectomy, which was quickly accepted by the professional oncogynecological community. The Querleu and Morrow (Q–M) classification of radical hysterectomy has provided a unique opportunity for uniform surgical and anatomical terminology. The classification offers detailed explanations of anatomical landmarks and resection margins for the three parametria of the uterus. However, there are still some disagreements and misconceptions regarding the terminology and anatomical landmarks of the Q–M classification. This article aims to highlight the surgical anatomy of all radical hysterectomy types within the Q–M classification. It discusses and illustrates the importance of anatomical landmarks for defining resection margins of the Q–M classification and reviews the differences between Q–M and other radical hysterectomy classifications. Additionally, we propose an update of the Q–M classification, which includes the implementation of parauterine lymphovascular tissue, paracervical lymph node dissection, and Selective-Systematic Nerve-Sparing type C2 radical hysterectomy. Type D was modified according to current guidelines for the management of patients with cervical cancer. The detailed explanation of the surgical anatomy of radical hysterectomy and the proposed update may help achieve surgical harmonization and precise standardization among oncogynecologists, which can further facilitate accurate and comparable results of multi-institutional surgical clinical trials.

## 1. Introduction

Cervical cancer (CC) ranks as the fourth most common cancer among women worldwide [1,2,3] and remains one of the leading causes of mortality in developing countries. In 2020, more than 604,000 women were diagnosed with CC, resulting in over 304,000 deaths [1,4]. Treatment of early-stage cervical cancer (CC) generally includes either simple or radical hysterectomy (RH) combined with pelvic lymph node dissection (PLND). Procedures such as large loop excision of the transformation zone, cold knife conization, or trachelectomy can be performed for patients desiring future fertility, depending on the disease stage and associated risk factors [5]. Advanced stages of CC are usually treated using chemoradiation therapy or, eventually, adjuvant radiation therapy [1]. Consequently, radical hysterectomy (RH) remains the “gold standard” treatment for patients with IA (selected), IB1, IB2, IB3 (selected), and IIA1 stages of CC [6].

Radical hysterectomy, introduced over a century ago, has been widely adopted by oncogynecologists worldwide [7,8]. The widespread use of RH techniques has been paralleled by the development of various surgical classifications of the procedure. While some of these classifications never gained broad recognition [9,10,11,12], others, such as the Piver–Rutledge–Smith classification published in 1974, became universally accepted [13]. In 2008, Querleu and Morrow proposed a novel classification of RH [14]. The Querleu–Morrow (Q–M) classification gained wide popularity and has since been cited in numerous publications. Its rapid adoption in the oncogynecological community suggests that most Q–M CC classification principles are properly selected. However, there are still some disagreements and misconceptions regarding the terminology and anatomical landmarks of the Q–M classification.

This article aims to highlight the surgical anatomy of all RH types within the Q–M classification. It discusses and illustrates the importance of anatomical landmarks for defining resection margins in the Q–M classification and reviews the differences between Q–M and other RH classifications. Furthermore, we propose an update of the Q–M RH classification.

### Discrepancies between Anatomical Nomenclature and Surgical Anatomy of the Parametrium

**Figure 1:**
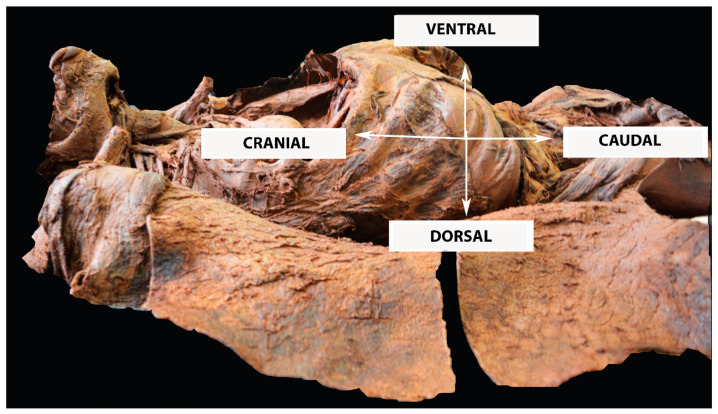
Spatial orientation in the surgical supine position. Medial and lateral spatial orientations are not shown but are also used in the present article (author’s own material—embalmed cadaver—dissection by author S.K.).

In this discussion, terms such as “cranial” versus “caudal” and “ventral” versus “dorsal” will be mainly used instead of “superficial” versus “deep” and “anterior” versus “posterior.” “Lateral” versus “medial” will also be incorporated instead of “external” versus “internal”. These terms, which define spatial orientation, were also used in the Q–M classification. However, the classification describes these terms in the sagittal plane [14,15]. Nevertheless, it is better to highlight these surgical-anatomical landmarks in the surgical supine position. If another position is discussed in the present article, it will be explained in the text. Additionally, Q–M stated that terms like “anterior”, “posterior”, “deep”, “superficial”, “external”, and “internal” are incorrect regarding spatial orientation [14]. However, it is important to note that these terms are in concordance with the majority of anatomical and surgical textbooks, and the rationale for their omission in the original article on RH remains somewhat unclear [12,16,17,18]. Spatial orientations used in the article are shown in Figure 1.

**Figure 2:**
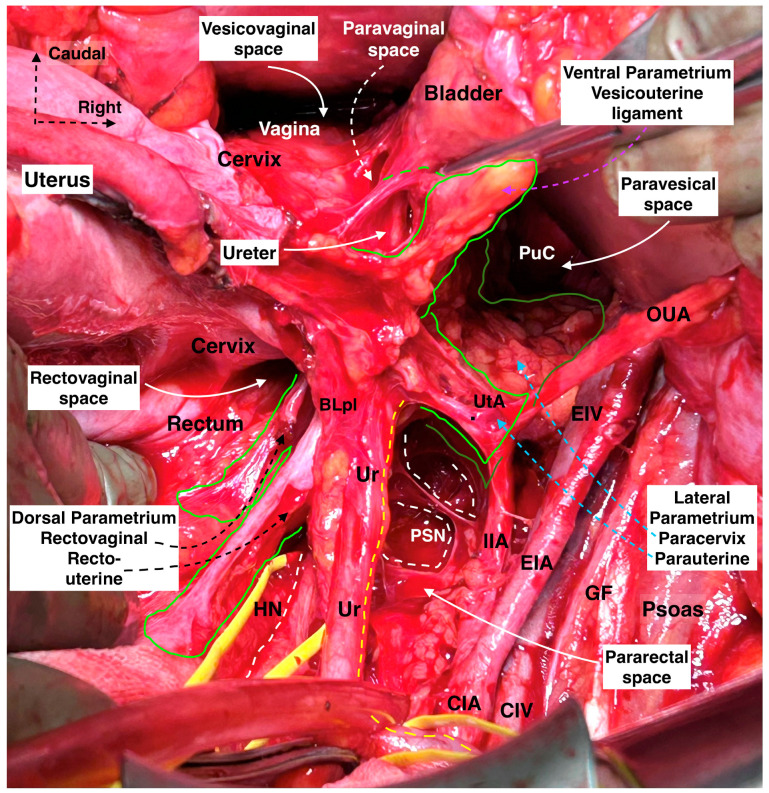
The parametrium: lateral, ventral, and dorsal paratissue of the uterus. (Open surgery; surgical dissection by author I.S.). The green lines show the limits of the parametrial tissue. The dashed green line shows the cleavage plane for the ventral parametrium and the paravaginal space dissection. The white dashed lines show the pelvic splanchnic nerves and the hypogastric nerve. The yellow dashed line shows the course of the ureter. BLpl: broad ligament posterior leaf, HN: hypogastric nerve, Ur: ureter, PSN: pelvic splanchnic nerves, PuC: pubococcygeus, UtA: uterine artery, IIA: internal iliac artery, OUA: obliterated umbilical artery, EIV: external iliac vein, EIA: external iliac artery, CIV: common iliac vein, CIA: common iliac artery, GF: genitofemoral nerve.

The term “parametrium” refers to the fatty lymphoid tissue around the uterus (including the uterine body and cervix) and defines the three parametria—the lateral, ventral, and dorsal parametria. These parametria represent the attachments of the uterine cervix to the surrounding organs (ventral and dorsal parametria) and the pelvic sidewall (lateral parametria) [17,18,19]. According to Ercoli et al., the term “parametrium” specifically defines the ventral portion of the cardinal ligament (located above the ureter) [20]. *Terminologia Anatomica* describes the “paracervix” as a component of the “parametrium”, and the cardinal ligament is denoted as an independent anatomical structure [21,22]. Etymologically, the term “metra” is the Latin word for “uterus”, and “para” means “close to” or “surrounding” [19]. Some editions of anatomical textbooks define the parametrium as the tissue passing to the sides of the cervix, laterally located between the two layers of the broad ligament [16]. Beyond these controversies, it is better to identify the parametrium as the paratissue of the uterus in lateral, ventral, and dorsal directions (Figure 2).

**Figure 3:**
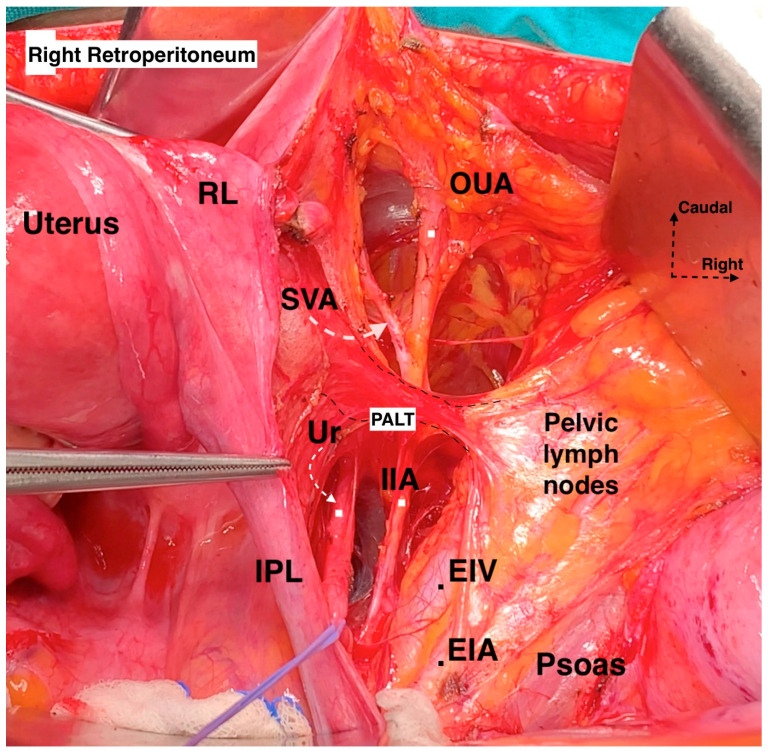
Parauterine lymphovascular tissue (PALT) (Open surgery; surgical dissection by author I.S.). EIV: external iliac vein, EIA: external iliac artery, IIA: internal iliac artery, OUA: obliterated umbilical artery, SVA: Superior vesical artery, Ur: ureter, IPL: infundibulopelvic ligament, RL: round ligament, PALT: parauterine lymphovascular tissue.

**Figure 4:**
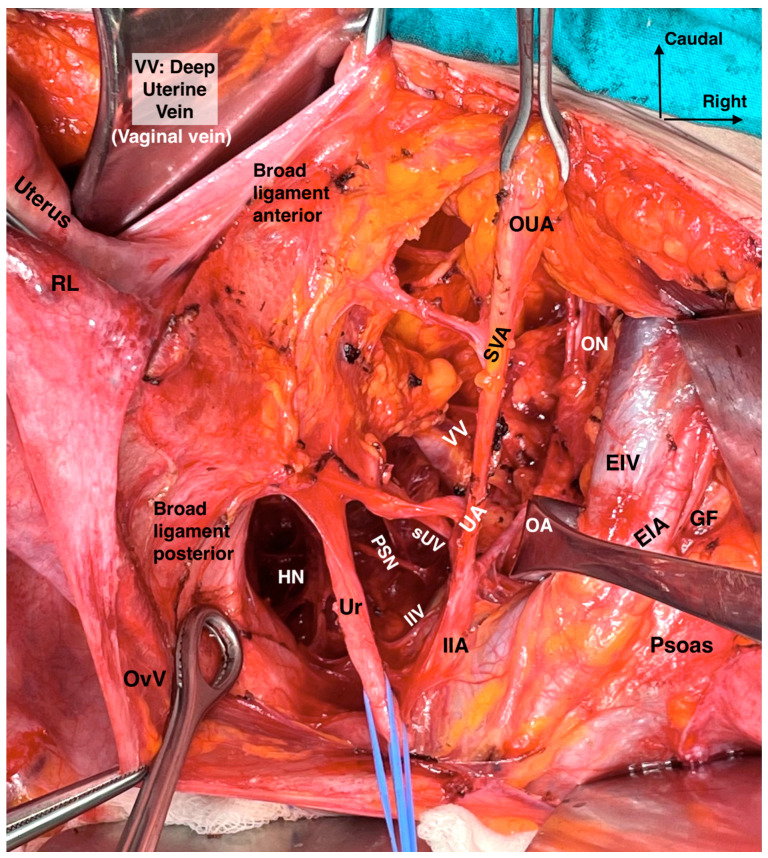
Vasculature of the lateral parametrium for parauterine (uterine artery and superficial uterine vein) and paracervix (deep uterine vein) tissue. (Open surgery; surgical dissection by author I.S.). EIV: external iliac vein, EIA: external iliac artery, GF: genitofemoral nerve, IIA: internal iliac artery, OUA: obliterated umbilical artery, UA: uterine artery, SVA: superior vesical artery, OA: obturator artery, ON: obturator nerve, IIV: internal iliac vein, sUV: superficial uterine vein, VV: vaginal vein, Ur: ureter, HN: hypogastric nerve, PSN: pelvic splanchnic nerves, OvV: ovarian vessels, RL: round ligament.

The lateral parametrium (LP) becomes visible after the dissection of the paravesical and pararectal spaces. The LP consists of the parauterine lymphovascular tissue (PALT)—a lymphatic tissue connecting the uterus with the lateral pelvic lymph nodes)—the uterine artery, the superficial uterine vein (ventral part, above the ureter), and the paracervix with the deep uterine vein (dorsal part, below the ureter) (Figure 3 and Figure 4). In the original description of the Q–M classification, the authors did not define or use the term “parauterine lymphovascular tissue” but only used the term “paracervix” to define the lateral attachments of the uterus [14]. Indeed, Querleu et al. stated that “lateral parametrium” is relevant to the paracervix [23]. The term “parauterine lymphovascular tissue” is not recognized by other authors and articles dedicated to RH, except for the recent RH articles of Querleu [24,25,26,27,28]. Following the article by Lührs et al. [29], Querleu used the terminology “parauterine” to define the ventral part of the LP [22,26,30]. The “parauterine tissue” is composed of the uterine artery, the superficial uterine vein, the PALT, and the fatty tissue. The PALT extends from the uterine body and lies between the broad ligament (medially), the obliterated umbilical artery (laterally), the superior vesical artery (ventrally), and the ureter (dorsally). The lymph channels and nodes form a direct connection between the uterus and the lateral pelvic sidewall. These lymphatic channels are parallel to the uterine artery and drain lymph from the uterus to the external iliac and obturator lymph nodes [29].

**Figure 5:**
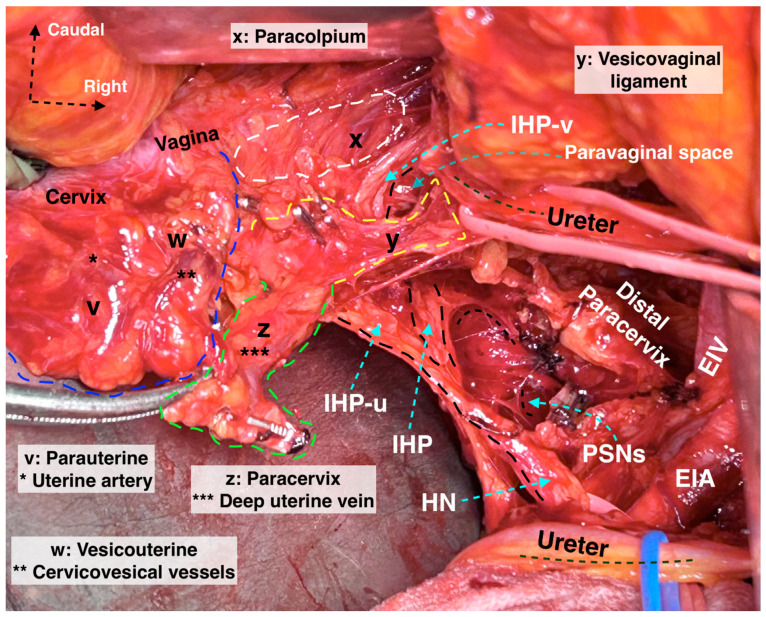
The paracolpium, vesicovaginal ligament, and paracervix with pelvic autonomic nerves. (Open surgery; surgical dissection by author I.S.). HN: hypogastric nerve, PSNs: pelvic splanchnic nerves, IHP: inferior hypogastric plexus, IHP-u: inferior hypogastric plexus uterine branches, IHP-v: inferior hypogastric plexus vesical branches, EIV: external iliac vein, EIA: external iliac artery.

The paracervix is located below (dorsal to) the ureter and extends from the uterine cervix–upper vagina to the dorsolateral part of the pelvic sidewall (ischial spine or proximal part of the tendinous arch of levator ani) [14,21,31]. The term “paracervix” replaces the old terms such as “cardinal ligament” or “Mackenrodt’s ligament”. The paracolpium defines the fatty-lymphoid tissue around the upper-middle vagina that consists of a network of blood vessels at the lateral aspect of the vaginal wall. In Gray’s Anatomy, the paracolpium is stated as the continuation of the parametrium along the vagina [16]. According to *Terminologia Anatomica* and the Q–M classification, the “paracolpium”, also referred to as “paracolpos”, is part of the paracervix, as there is no clear anatomical boundary between the lateral attachments of the supravaginal uterine cervix and the upper vagina [14,16]. This unification of terminology could introduce ambiguity among oncogynecologist. However, recent clarification from Querleu et al. suggests that this anatomical unity (“paracolpium” as part of the “paracervix”) refers only to the upper third of the vagina, as the paracolpium could be identified as an independent anatomical structure at the middle third of the vagina—lateral and dorso-caudal to the vaginal fornices [12,26]. In contrast, the term “paracolpium” is an integral part of many Japanese articles dedicated to RH [32,33]. Yabuki et al. defined the paracolpium as a neurovascular structure situated on the lateral aspect of the vagina and covered by the vaginal fascia. Lateral and caudal to the paracolpium, the tendinous arch of the pelvic fascia is identified [32]. Fujii et al. stated that the paracolpium represents the lateral paravaginal tissue, which contains the blood vessels of the vaginal wall. The authors termed it “vaginal blood vessels”. These vessels lie parallel to the lateral vaginal wall and drain into the deep uterine vein (vaginal vein). The vesical nerve branches of the inferior hypogastric plexus (IHPvb) are parallel to the paracolpium and attach to its lateral border [33]. According to Fujii et al., lateral to the paracolpium, Obakayashi’s paravaginal space can be dissected. The paracolpium, together with the vaginal blood vessels and vesical nerve branches, represents the medial limit of the space, whereas the vesicovaginal ligament is the lateral limit [33]. Some European authors also make a distinction between the paracolpium and the paracervix [12,28,34]. Selcuk stated that the paracolpium could be encountered in the upper 1/3–1/2 of the vagina, and it is composed of vascular-rich fatty-lymphoid tissue identified only after dissection and lateralization of the distal ureter from the vagina (Figure 5) [34].

**Figure 6:**
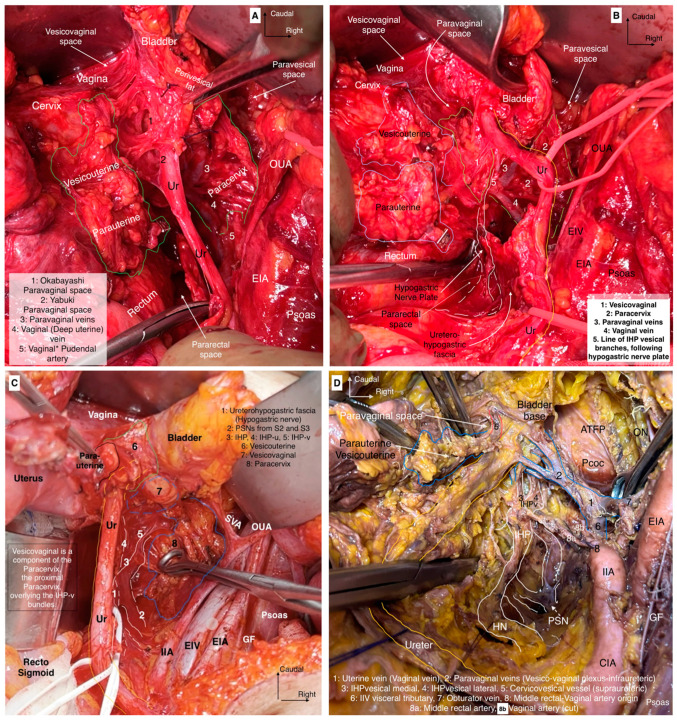
(**A**): Paravaginal spaces (Okabayashi and Yabuki) dissection (*) and (**B**): vesicovaginal ligament, paravaginal veins-vesicovaginal veins, paracervix, and vaginal vein. Associations with the pelvic autonomic nerves. (**C**): Medial (proximal) and lateral (distal) paracervix. The vesicovaginal ligament lies ventral to the inferior hypogastric plexus vesical branches. (Surgical dissection by author I.S.). (**D**): Vascular and nervous portion of the paracervix. Paravaginal veins (vesicovaginal veins) with the vaginal vein, and the pelvic autonomic nerve plate. (Open surgery; cadaveric dissection by author IS). EIV: external iliac vein, EIA: external iliac artery, GF: genitofemoral nerve, IIA: internal iliac artery, OUA: obliterated umbilical artery, SVA: superior vesical artery, Ur: ureter, PSNs: pelvic splanchnic nerves, IHP: inferior hypogastric plexus, IHP-v: inferior hypogastric plexus vesical branches, IHP-u: inferior hypogastric plexus uterine branches, IIV: internal iliac vein, HN: hypogastric nerve, Pcoc: pubococcygeus, ATFP: arcus tendineus fascia pelvis, ON: obturator nerve, IIV: internal iliac vein, CIA: common iliac artery.

Okabayashi’s paravaginal space (medial limit—paracolpium, vesical nerve branches, and lateral limit—vesicovaginal ligament) can be encountered at the ureterovesical junction by making a dissection between the lateral vaginal wall and the distal ureter after total transection of the vesicouterine ligament [24,28,33]. On the other hand, Yabuki also defined a space that is 1–1.5 cm cranial to the level of Okabayashi’s paravaginal space, developed by performing a dissection between the lateral vaginal wall and distal ureter just after the transection of the parauterine tissue and medial mobilization of the half part of vesicouterine ligament [28,32,33]. Developing Okabayashi’s paravaginal space provides total lateralization of the distal ureter and access to the entire vesicovaginal ligament and paracervix. Developing Yabuki’s paravaginal space provides access to the proximal vesicovaginal ligament and proximal parts of the vesical nerve branches (Figure 6).

**Figure 7:**
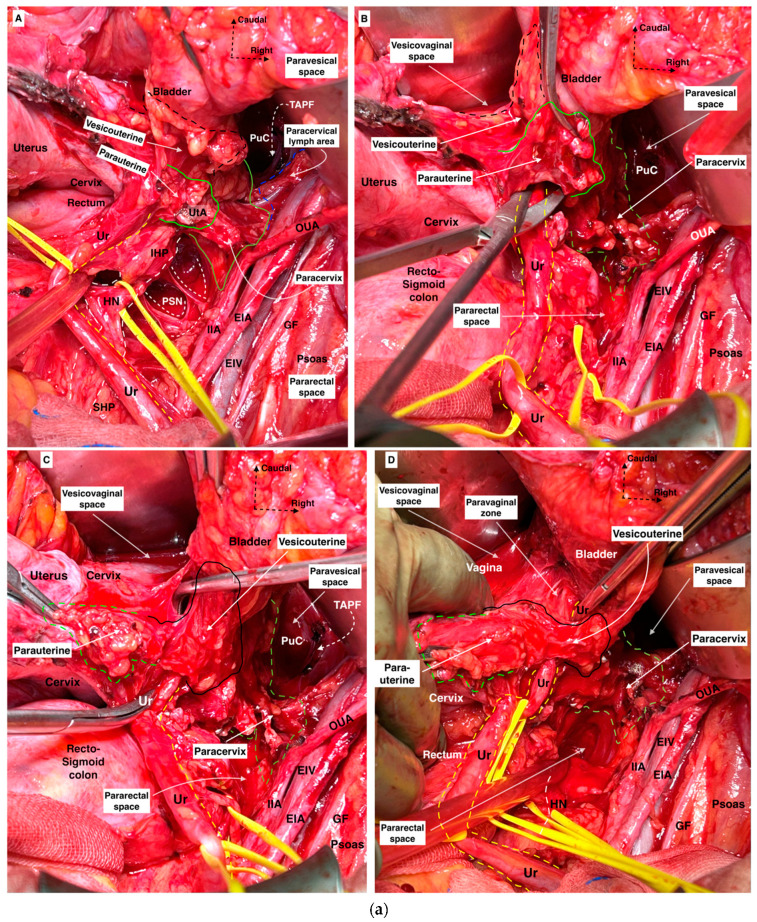

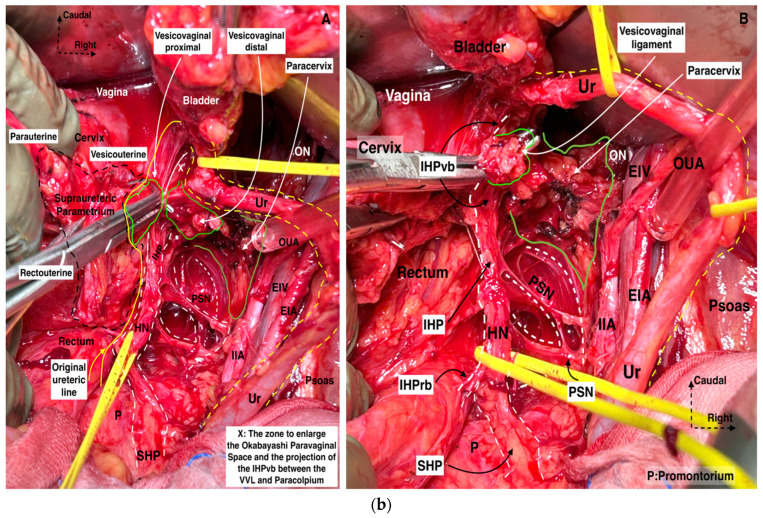
(**a**): (**A**): Lateral parametrium, (**B**): parauterine, ventral to the ureter, and paracervix, dorsal to the ureter, and (**C**,**D**): ventral parametrium dissection after craniomedial mobilization of the parauterine tissue. There are clear (avascular) dissection pathways and ureteric tunnel dissection at the 1 and 11 o’clock positions of the distal ureter. For the right side, the 1 o’clock position provides a more lateral approach with extended dissection of the vesicouterine ligament, and the 11 o’clock position provides a medial approach with limited dissection of the vesicouterine ligament. (Mirror image for the left ureter) (Open surgery; surgical dissection by author I.S.). (EIV: external iliac vein, EIA: external iliac artery, IIA: internal iliac artery, OUA: obliterated umbilical artery, UtA: uterine artery, GF: genitofemoral nerve, Ur: ureter, SHP: superior hypogastric plexus, HN: hypogastric nerve, PSN: pelvic splanchnic nerves, IHP: inferior hypogastric plexus, PuC: pubococcygeus, TAPF: tendinous arch of pelvic fascia). (**b**): (**A**,**B**): Developing the paravaginal space and total ureteric lateralization is the key to reaching the vesicovaginal ligament, inferior hypogastric plexus vesical branches, and the entire paracervix. (Open surgery; surgical dissection by author IS). EIV: external iliac vein, EIA: external iliac artery, IIA: internal iliac artery, OUA: obliterated umbilical artery, Ur: ureter, ON: obturator nerve, SHP: superior hypogastric plexus, HN: hypogastric nerve, PSN: pelvic splanchnic nerves, IHP: inferior hypogastric plexus, IHP-vb: inferior hypogastric plexus vesical branches, IHP-rb: inferior hypogastric plexus rectal branches, VVL: vesicovaginal ligament.

The paracervix is divided into two parts in a transverse (horizontal) plane—medial and lateral (the demarcation line between the two parts is the ureter). The medial part, located close to the cervix, is composed of condensed fibrous tissue, whereas the lateral part contains soft lymph-bearing tissue (where lymph nodes can be encountered) surrounding vessels and nerves [14] (Figure 7a). Indeed, dividing the transverse plane according to the hypogastric nerve (HN) plate will also be used because the ureter and the HN plate lie on the same sheet. Therefore, ureteric dissection and lateralization may not alter the surgical plane for distal paracervix. Moreover, in a longitudinal (vertical) plane, the paracervix is divided by the vaginal vein (deep uterine vein) into a ventral-vascular part and a dorsal-nervous part. The vascular part contains the inferior vesical and vaginal arteries, whereas the nervous part consists of the inferior hypogastric plexus (IHP) with the distal portion of the pelvic splanchnic nerves [14,23,35] (Figure 6).

The ventral parametrium is identified after the dissection of the medial paravesical space (lateral limit: umbilical ligament; medial: vesicouterine ligament) and the vesicouterine septum (vesicouterine/vesicovaginal spaces). The ventral parametrium is composed of the vesicouterine (ventral part) and vesicovaginal (dorsal part) ligaments. The vesicouterine ligament (VUL) is located above and medial to the ureter (ventromedial) (Figure 7a). The ligament extends between the anterolateral part of the cervix and the bladder. The VUL becomes clearly identifiable by the craniomedial mobilization of the uterine artery and superficial uterine vein after transecting them at the level of the ureter or lateral to the ureter, close to the internal iliac artery [14,15,22,23] (Figure 7a). The superficial vesical vein and the cervicovesical vessels are part of the VUL [24].

The vesicovaginal ligament (VVL) is located below and lateral to the distal ureter (dorsolateral). This ligament is only identified after the transection of the VUL and the development of Okabayashi’s paravaginal space. The VVL has no anatomic connection with the uterine corpus and lies between the upper vagina and bladder base at the caudal part of the ureterovesical junction (Figure 7b). Therefore, the term “posterior leaf of the vesicouterine ligament” is not accurate in defining this fatty-lymphoid tissue [24]. The VVL is situated laterally to the vagina and between Okabayashi’s paravaginal space and the medial paravesical space. The VVL consists of paravaginal veins, which could also be termed “vesicovaginal veins” (venous vessels draining blood from the vesical to the uterovaginal venous plexus), that drain into the vaginal vein [16,17,24] (Figure 6). The vaginal vein is synonymous with the deep uterine vein [12,15]. The paravaginally located vesicovaginal veins are also referred to as the middle and inferior vesical veins [24] or the medial and lateral vesical veins [25]. According to Querleu et al., the VVL also contains the vesical nerve branches of the IHP [26]. However, other authors reported that vesical nerve branches are not part of the VVL [24,27]. Sekiyama et al. divided the paracervical tissue, which is on the dorsal side of the distal ureter and lateral to the upper vagina, into medial (paracolpium) and lateral (VVL) parts. The authors reported that vesical nerve branches lie on the lateral part of the vagina and intertwine with the paracolpium (the medial portion of the paracervix) [27]. Zapardiel et al. [28] and Muallem et al. [25] also reported that vesical nerve branches are not part of the VVL. A recent European paper reported that the vesical branches of the IHP lie between the VVL and the paracolpium, and the VVL is located at the ventral part of the vesical nerve branches [18].

**Figure 8:**
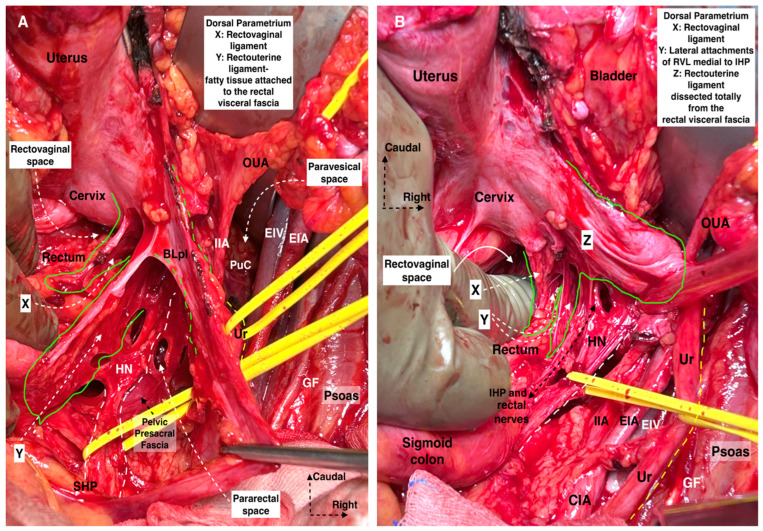
(**A**,**B**): Dorsal parametrium: rectouterine and rectovaginal ligament. (Open surgery; surgical dissection by author I.S.). EIV: external iliac vein, EIA: external iliac artery, IIA: internal iliac artery, OUA: obliterated umbilical artery, Ur: ureter, GF: genitofemoral nerve, CIA: common iliac artery, HN: hypogastric nerve, IHP: inferior hypogastric plexus, RVL rectovaginal ligament, SHP: superior hypogastric plexus, PuC: pubococcygeus, BLpl: broad ligament posterior leaf.

The dorsal parametrium is identified after the dissection of the medial pararectal space (Okabayashi’s space) and the rectovaginal septum (rectovaginal space in classical terminology) (Figure 8). The dorsal parametrium is represented by the rectouterine ligament (ventral part) and the rectovaginal ligament (dorsal part) [15,23,34]. The rectovaginal ligament lies in the deeper part of the dorsal parametrium between the dorsolateral part of the upper vagina and the pelvic parietal fascia covering the anterior part of Sacral 2–4 level (ventral nerve rami of the sacral roots). It is thicker and stronger compared to the rectouterine ligament and provides stabilization to the upper vagina and pelvic support mechanism [34]. On the one hand, the fatty tissue between the dorsolateral part of the cervix and the rectum is a connective tissue that has no suspensory mechanism and is covered by the rectouterine peritoneum called the rectouterine ligament. On the other hand, to reach the rectovaginal ligament, the rectouterine ligament should be dissected and transected. Querleu et al. defined the ventral part of the dorsal parametrium—the uterosacral ligament—and stated that it is not a true ligamentous structure [22]. Therefore, in the last consensus article on RH classification, only the resection line of the dorsal part of the dorsal parametria (rectovaginal ligament) was described [26]. The majority of authors agree with the term “rectovaginal ligament” for the dorsal part of the dorsal parametrium [15,19,27,32,34,35,36]. In concordance with the last update of the Q–M RH classification, we will mainly describe the resection line of the rectovaginal ligament and state the surgical role of the rectouterine ligament.

The mesoureter is a mesentery-like sheet of connective tissue that surrounds the ureter, the hypogastric nerves, and the proximal part of the IHP. The term ureterohypogastric fascia is also used to describe this anatomical structure. The development of Okabayashi’s pararectal space (medial—rectovaginal ligament; lateral—ureter, mesoureter, hypogastric nerve) allows for safe dissection and lateralization of the ureter. Dissection of the mesoureter provides identification of the hypogastric nerve, thus facilitating the dissection of the pelvic autonomic nerve plate. The mesoureter is located dorsally to the ureter. One might suggest using the term “posterior mesoureter” since some authors use the term “anterior mesoureter” to define the VVL [32]. The term “anterior mesoureter” is also used in surgical resection-based ontogenetic cancer field theory for CC [37].

## 2. Parametrium and Pelvic Autonomic Nerve System

**Figure 9:**
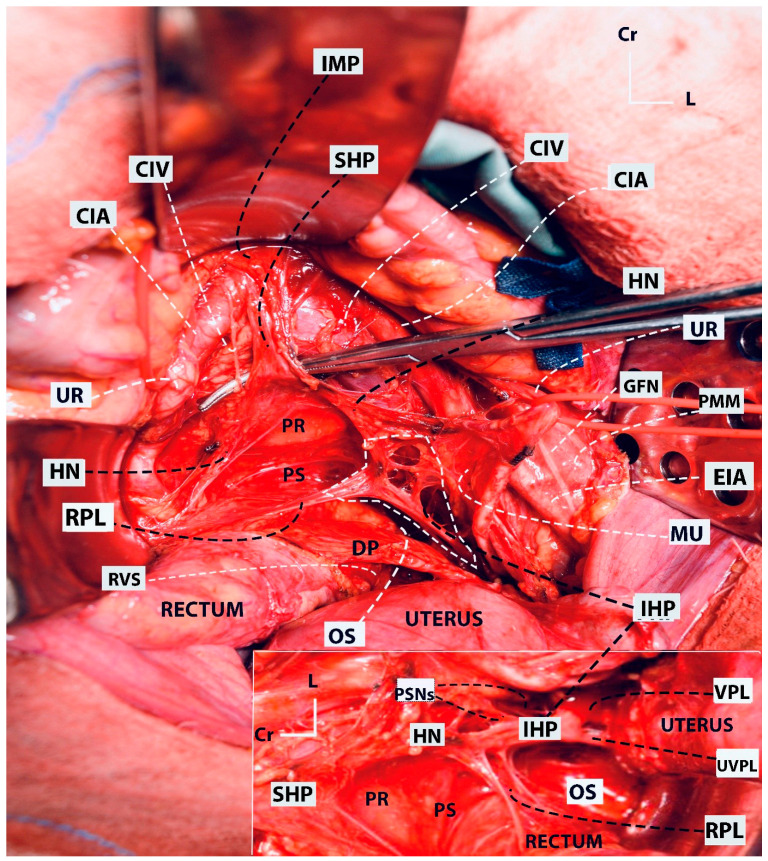
Superior and inferior hypogastric plexus in the female pelvis (Open surgery; surgical dissection by authors Y.K. and S.K.). In the right corner, the inferior hypogastric plexus is shown in optical magnification. IMP—inferior mesenteric plexus; SHP—superior hypogastric plexus; CIV—common iliac vein; CIA—common iliac artery; UR—ureter; HN—hypogastric nerve; RPL—rectal nerve plexus; PR—promontory; PS—ventral part of the presacral space; DP—dorsal parametrium; OS—Okabayashi’s pararectal space; IHP—inferior hypogastric plexus; PSNs—pelvic splanchnic nerves; VPL—vesical nerve plexus; UVPL—uterovaginal nerve plexus; MU—mesoureter; EIA—external iliac artery; PMM—psoas major muscle; GFN—genitofemoral nerve; Cr—Cranial; L—left.

The superior hypogastric plexus (SHP), hypogastric nerves, pelvic splanchnic nerves (PSNs), IHP, rectal–uterovaginal–bladder nerve branches of the IHP are elements of the Pelvic Autonomic Nerve System (PANS). The PANS is an indispensable part of the parametrial anatomy and surgical approach in radical hysterectomy (Figure 9).

The thoracolumbar splanchnic nerves (Thoracal 10—Lumbar 2), with the contribution of the Lumbar 3 splanchnic nerve, form the SHP at the caudal part of the inferior mesenteric artery. The SHP mainly carries sympathetic innervation and lies ventral to the aortic bifurcation and the left common iliac vein.

At approximately the level of the promontory, the SHP divides into the left and right HN, which conveys sympathetic innervation to the pelvis. Initially, the HN lies dorsolateral to the rectum in the ventrolateral part of the presacral fascia and later runs along the lateral wall of the rectum, which corresponds to the lateral part of the rectouterine ligament in the medial pararectal space (Okabayashi’s pararectal space). The HN runs 2 cm dorsal to the ureter within the same fascial sheet, called the mesoureter.

The PSNs arise from the Sacral 2–4 roots (ventral rami of the sacral roots) and carry parasympathetic innervation. The PSNs pierce the piriformis muscle, lie in the dorsolateral part of the lateral pararectal space (close to the internal iliac vein or its visceral tributary), and run obliquely towards the medial side, the caudal part of the medial pararectal space, within the dorsal part of the paracervix tissue and are dorsal to the level of the deep uterine vein. The PSNs (indeed the PANS) are located medial to the internal iliac vascular system.

The HN and PSNs merge and form the inferior hypogastric plexus (IHP) at the medial aspect of the paracervix and dorsal to the deep uterine vein (vaginal vein), lateral to the rectovaginal ligament and rectum, and dorsolateral to the upper vagina. The IHP is a mixed ganglion, the pelvic ganglion, and conveys sympathetic and parasympathetic innervation.

**Figure 10:**
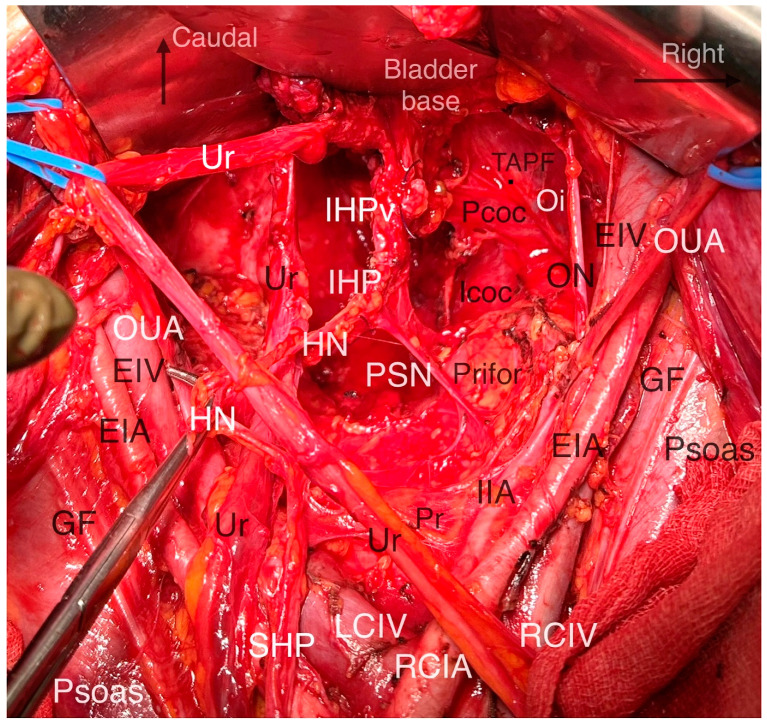
Pelvic Autonomic (Hypogastric) Nerve System by the superior hypogastric plexus, hypogastric nerve, pelvic splanchnic nerves, inferior hypogastric plexus, and bladder nerve branches of the inferior hypogastric plexus. (Open surgery; surgical dissection by author I.S.). Selective-Systematic Nerve-Sparing Radical Hysterectomy (unilateral systematic nerve-sparing) plus low anterior resection for an endometrial cancer case where there is a continuous infiltration of the parametrium and rectal involvement on the left side but clear parametrial margins at the right side. EIV: external iliac vein, EIA: external iliac artery, IIA: internal iliac artery, OUA: obliterated umbilical artery, Ur: ureter, GF: genitofemoral nerve, RCIA: right common iliac artery, RCIV: right common iliac vein, LCIV: left common iliac vein, ON: obturator nerve, Oi: obturator internus muscle, Pcoc: pubococcygeus, Icoc: iliococcygeus, Prifor: piriformis, TAPF: tendinous arch of pelvic fascia, Pr: promontorium, SHP: superior hypogastric plexus, HN: hypogastric nerve, PSN: pelvic splanchnic nerves, IHP: inferior hypogastric plexus, IHP-v: inferior hypogastric plexus vesical branches.

The vesical branches of the IHP run towards the bladder base at the dorsolateral aspect of the distal ureter and the ureterovesical junction and lateral to the upper vagina. The IHP vesical branches carry parasympathetic innervation for bladder functions (voiding), which contract the detrusor muscle and relax the internal urethral sphincter. The vesical branches of the IHP lie between the VVL and the paracolpium part of the paracervix. Their preservation constitutes the most crucial step in nerve-sparing radical hysterectomy. The VVL can be dissected at the ventral part of the vesical branches. The vesical nerve branches are intertwined with the paravaginal veins, which drain into the vaginal vein (Figure 6 and Figure 10).

### Pelvic Autonomic Nerve System and Possible Injury Areas with Respect to Cervical Cancer Surgery

- SHP—Low paraaortic lymphadenectomy
- HN—Rectouterine ligament dissection
- PSNs—Lateral paracervix dissection and medial paracervix dissection
- IHP—Medial paracervix dissection and rectovaginal ligament dissection
- Vesical branches of the IHP—VVL dissection and medial paracervix dissection near the paracolpium

## 3. Types of Radical Hysterectomy According to the Querleu and Morrow (Q–M) Classification

The first article on the Q–M classification mainly described the resection lines of the parametrial tissue in a transverse (horizontal) plane [14]. Cibula et al. further added the 3D anatomical template of the Q–M classification, which describes the resection limits of the three uterus parametria in three dimensions [15]. Moreover, it clearly outlines the resection lines of the three parametria in the longitudinal (vertical/deep) plane [15], whereas older classifications mainly highlight the transverse (horizontal) plane of transection [10,11,13]. Therefore, a comparison between classifications in a scheme or table is not applicable. On the other hand, the resection lines for types A and D RH described by Cibula et al. were not in total concordance with the first article on the Q–M classification [15]. Later, Querleu et al. introduced an update on the Q–M classification, incorporating the three-dimensional resection lines [23]. Recently, an international expert consensus was initiated to define and interpret the update of the Q–M classification [26]. The Q–M classification introduced clear anatomical margins for RH, which were modified according to Terminologia Anatomica. Therefore, the intervariability of the procedure among surgeons decreased, and RH arose to a more reproducible surgical approach. Furthermore, the integrity of pelvic autonomic nerves was included to clarify the nerve-sparing approach [12,23,26].

- Radical Hysterectomy Types and Summary of Parametrium

**Figure 11:**
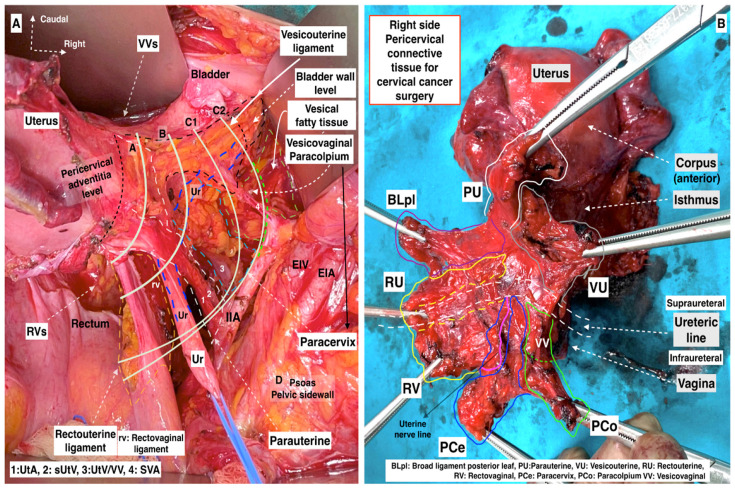
(**A**): Querleu–Morrow radical hysterectomy levels based on parametrial anatomy and the anatomical landmarks of pericervical adventitia, ureter, internal iliac artery, and pelvic sidewall. (**B**): Right side of the 3 parametria for cervical cancer surgery, which are divided into ventral and dorsal parts according to the longitudinal axis of the ureter. Indeed, paracolpium is a part of the paracervix. (Open surgery; surgical dissection by author I.S.). VVs: vesicovaginal space, RVs: rectovaginal space, Ur: ureter, EIV: external iliac vein, EIA: external iliac artery, IIA: internal iliac artery, UtA: uterine artery, sUtV: superficial uterine vein, UtV/VV: deep uterine vein/Vaginal vein, SVA: superior vesical artery, BLpl: broad ligament posterior leaf, PU: parauterine, VU: vesicouterine, RU: rectouterine, RV: rectovaginal, PCe: paracervix, PCo: paracolpium, VV: vesicovaginal.

The Q–M RH classification is primarily based on the transverse levels of pericervical adventitia (Type A), ureter (Type B), internal iliac artery (Type C), and pelvic sidewall (Type D). The paratissue of the uterus, parametrium, can be divided into ventral and dorsal parts with respect to the ureteric line. Therefore, the parametrial tissue that is excised during RH can be easily understood (Figure 11).

- Supraureteric Parametrium/Ventral Part of the Parametrium:
  - Ventral: Vesicouterine
  - Lateral: Parauterine
  - Dorsal: Rectouterine
- Infraureteric Parametrium/Dorsal Part of the Parametrium:
  - Ventral: Vesicovaginal
  - Lateral: Paracervix (the medial aspect adjacent to the upper-middle vagina, called paracolpium, indeed a part of paracervix)
  - Dorsal: Rectovaginal

### 3.1. Type A RH—Minimal Radical Hysterectomy

The aim of Type A RH is the total removal of the uterine cervix, which includes the pericervical adventitia (fascia), with a clear margin dorsal to the level of the vaginal fornices. Therefore, Type A RH is mainly formed by the minimum resection of the paratissue surrounding the uterine cervix.

In the transverse plane, the paracervix is transected medial to the ureter and lateral to the pericervical fascia (resection halfway between the ureter and the cervix), whereas in the longitudinal plane, the paracervix is resected at the dorsal part of the intravaginal portion of the uterine cervix where the lateral vaginal fornix is reached and opened [26,29]. The rectovaginal ligament and the VUL are resected at a distance from the uterus, minimally—5 mm resection of the ligaments [23,26]. The rectovaginal ligament is removed minimally to mobilize the upper vagina and provide a clear resection margin at the vaginal fornices. The VUL is removed minimally to lateralize the distal portion of the ureter, and the ureter is rolled to a safe area, thus maintaining a clear resection margin for the anterior vaginal fornices. Querleu et al. mentioned only the resection of the dorsal part of the dorsal parametrium—the rectovaginal ligament—as they stated that the uterosacral ligament (rectouterine ligament) is not a true ligamentous structure [22,26]. On the other hand, the rectouterine ligament is dissected and resected minimally close to the uterine cervix. Minimal removal of the three parametria was mentioned in the update to the Q–M classification [23]. The ureters are not unroofed during open or laparoscopic surgery but are just identified after minimal dissection of the ureteral tunnel, which provides limited lateralization to the ureter and prevents any thermal injury during the operation. Identifying the course of the ureter through the ureteral tunnel and partial dissection of the tunnel could be necessary in order to obtain minimal resection of the VUL. Additionally, resection of the rectovaginal ligament necessitates the identification and lateralization of the ureter by developing the medial pararectal space (Okabayashi’s pararectal space), as the ligament is an infraureteric retroperitoneal anatomical structure. The VVL and the paracolpium are not dissected and removed. Generally, less than 10 mm of the vagina is removed. The length of the resected vagina could be considered overtreatment in some patients, but it can help avoid positive margins or incomplete transection of the cervical stroma [23,26]. The pelvic autonomic nerves are intact, as the dissection and resection lines are ventral to the nerves. Type A RH requires partial dissection of the lateral pararectal and paravesical spaces (in order to remove the PALT), but it is not necessary to entirely dissect these spaces until their dorsal limits. During vaginal surgery, the position of the ureter is determined through palpation [14,23].

The first article on the Q–M classification reported only the transection of the paracervix in a transverse plane [14]. Transection of the PALT, ventral, and dorsal parametria were not mentioned in the first two articles on the Q–M RH classification [14,15]. It was only stated that the ventral and dorsal parametria are not resected at a distance from the uterus [14]. However, the update of the classification introduced resection lines for the three parametria during Type A RH [23]. The aim of Type A minimal RH is to ensure the removal of the pericervical fascial tissue to the level of the vaginal fornices, along with the corresponding vaginal tissue. Therefore, it is noteworthy that Type A RH is not a simple extrafascial hysterectomy. It is more radical than a simple extrafascial hysterectomy and less radical than type B RH.

#### 3.1.1. Indications for Type A RH Are as Follows [23]

- Stage IA CC (selected cases with risk factors and patients who do not desire future fertility)
- Low-risk stage IB1 CC (cervical tumor ≤ 2 cm, absence of lymphovascular space invasion, absence of deep stromal invasion, absence of metastatic pelvic lymph nodes)
- In rare cases, as a final procedure after neoadjuvant chemoradiation (or radiation or chemotherapy alone) or primary chemoradiation due to advanced CC
- Application in future clinical trials

#### 3.1.2. During Type A Minimal RH, the Resection Lines of the Three Parametria Are as Follows [14,15,23,26]

1. Ventral parametria
  - Transverse: The VUL is minimally resected close to the uterine cervix. The ureteric tunnel is not totally dissected, and the distal ureter is not unroofed.
  - Longitudinal plane: The VVL is not transected.
2. Lateral parametria
  - Transverse plane: The paracervix is transected medial to the ureter and lateral to the pericervical fascia. The PALT is removed separately.
  - Longitudinal plane: The resection extends caudally to the level of the vaginal fornices at the medial edge of the ureteric line.
3. Dorsal parametria
  - Transverse plane: The rectovaginal ligament is minimally transected close to the posterior vaginal fornix.
  - Longitudinal plane: The transection level does not extend more caudally than the the vaginal fornices.

#### 3.1.3. Comments

Other classifications used the term “simple extrafascial hysterectomy” to define the first class or type of their RH classifications [11,12,13]. The corresponding term “extrafascial” actually describes a surgical dissection that provides complete removal of the cervical stroma. Therefore, “extrafascial” is equal to a “simple” hysterectomy, which does not include the removal of any paracervical or parauterine tissue [23,26]. On the other hand, intrafascial hysterectomy, also known as the Aldridge procedure, is indicated only for benign gynecologic diseases [38,39]. Intrafascial hysterectomy preserves the anatomic connection between the vagina and endopelvic fascia [38,39], where the cervical stroma is not resected totally, along with the outer pericervical fascial margin. During Type A RH, the upper, anterior, paracervical lymphatic pathway (PALT), as described by Lührs et al. [29], containing lymphatics and possible metastatic nodes, can be transected totally from the internal iliac artery/pelvic parietal lymph node level. In some cases, mainly in patients with lower-normal body mass index, the PALT could be identified and resected separately without transection of the uterine artery/superficial uterine vein at the level of the internal iliac vessels (Figure 2). Type A RH, according to the Q–M classification, was not mentioned in the SHAPE trial, which we consider a limitation, as one of the main purposes of the classification was the application in future clinical trials [40].

### 3.2. Type B Radical Hysterectomy

Type B RH is divided into two subtypes: Subtype B1, which can be referred to as “modified” radical hysterectomy, and subtype B2, which builds upon B1 by adding paracervical lymphadenectomy. The main resection line of the parametria for Type B RH is the axis of the ureter.

#### 3.2.1. Type B1 RH—Modified Radical Hysterectomy

This type of RH corresponds to the “modified” RH by Wertheim. The ureter is unroofed and lateralized in order to transect the parauterine and paracervix tissue at the level of the ureteral tunnel. The ureter is laterally dissected from the ureteral tunnel to achieve partial resection of the VUL [14,23].

In order to clearly identify the VUL, the medial paravesical space, and the vesicovaginal septum should be dissected. The lateral pararectal and paravesical spaces are also developed. In this subtype, identification of the pelvic autonomic nerves is not necessary because the dissection and resection lines are ventral to the pelvic autonomic nerves [14,15,23,26]. So, the pelvic autonomic nervous system is left untouched, and it is preserved. However, aggressive use of thermal energy may injure the PANS. An exception is the identification and lateralization of the HN (through the development of Okabayashi’s pararectal space) during the partial resection of the rectovaginal ligament. Moreover, care should be taken with the proximal part of the IHP. Consequently, in Type B1 RH, we develop four lateral avascular spaces: the lateral and medial paravesical and pararectal spaces. Zapardiel et al. stated that Yabuki’s paravaginal space can be developed during modified RH [28]. Developing the paravaginal space facilitates the identification of the vesicovaginal venous plexus and may decrease bleeding during ventral parametria dissection. Approximately 10 mm of the vagina is removed.

##### During Type B1 RH, the Resection Lines of the Three Parametria Are as Follows [15,19,23,26]

1. Ventral parametria
  - Transverse: Partial resection of the VUL is performed—halfway between the urinary bladder and uterus.
  - Longitudinal plane: The VVL is not resected, and the vesical nerve branches are left untouched.
2. Lateral parametria
  - Transverse plane: The parauterine and paracervix tissue is resected at the level of the ureter (at the level of the ureteral tunnel).
    As an additional mark, the parauterine tissue can be transected at the level of the IIA above the ureter in order to remove the PALT. If possible, PALT could be removed separately, without transection of the uterine artery/superficial uterine vein at the level of the internal iliac vessels.
  - Longitudinal plane: The resection line of the paracervix depends on the longitudinal plane of the vaginal cuff resection.
3. Dorsal parametria
  - Transverse plane: Partial resection of the rectovaginal ligament occurs after identification and lateralization of the ureter, mesoureter, and HN. The resection is performed halfway between the rectum and the uterus.
  - Longitudinal plane: This depends on the resection plane of the vagina. The resection length is comparable to the amount of paracervix removed. The IHP should be spared during the excision of the longitudinal plane of the rectovaginal ligament.

##### Comments

**Figure 12:**
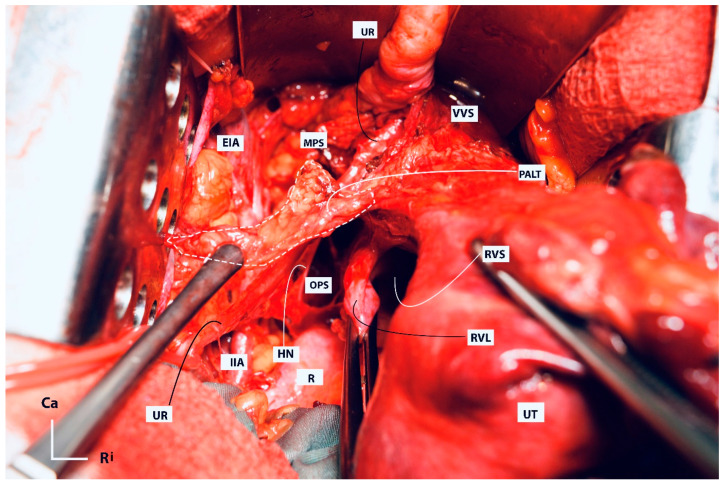
Resection of the parauterine lymphovascular tissue during B1 radical hysterectomy. (Open surgery; surgical dissection by authors Y.K. and S.K.). UR—ureter; RVS—rectovaginal space; OPS—Okabayashi’s pararectal space; HN—hypogastric nerve, R—rectum; IIA—internal iliac artery; UT—uterus; RVL—rectovaginal ligament; PALT—parauterine lymphovascular tissue; VVS—vesicovaginal space; MPS—medial paravesical space; EIA—external iliac artery Ri—right; Ca—caudal.

According to the original definition of Type B1 RH, the lateral parametrium is resected at the ureteric level [14,15], which implies that approximately half of the distal part of the PALT would not be removed. So, one might suggest adding the total resection of the PALT in Type B1 RH. Future incorporation of the PALT in Type B1 RH should be considered (Figure 12). Querleu has already highlighted the proportion of possible lymphatic metastases in the parauterine tissue, even in the early stages of CC [30].

The terminology “resection medial to the ureteral bed” does not correctly imply resection of the paracervix in Type B1 RH [14,23], as medial could be at any transverse level or even close to the cervix. Therefore, the resection of the paracervix at the level of the ureteral tunnel is more accurate in terms of defining resection lines.

In conclusion, for Type B RH, the resection lines of the tree parametria (except for the resection of PALT) do not expand laterally to the ureter. The Muallem classification for Type II RH involves the total removal of the ventral portions of the three paratissue of the uterus: the VUL is ligated at the ureteral junction to the bladder (the ureter should be completely dissected from the VUL), the rectouterine ligament is resected at the level of the rectum, and the parauterine tissue is resected at the level of the internal iliac vessels. Muallem defines all ventral parts of the three paratissue of the uterus as the “parametrium” and the dorsal parts as the “paracolpium”. The dorsal parts of the three paratissue of the uterus are not resected during Type II RH Muallem classification. The length of vaginal resection is 10–20 mm [12]. The Piver–Rutledge–Smith classification is equal to Type B (Q–M), except for the length of vaginal removal, which resects the upper one-third of the vagina, and the resection of PALT, in which the parauterine lymphovascular tissue is not resected [13]. The EORTC (Mota) classification, in turn, differs from the Q–M classification, as the ureters are dissected up to the point where they enter the bladder; the resection of the PALT is not mentioned, and the length of vaginal resection is more extensive than 1–2 cm [11].

During Type B1 RH, developing the paravaginal space partially (Yabuki’s space) will provide a safe excision plane for the VUL and upper vagina while adjusting the vaginal length by dissection and lateralization of the distal ureter without excision of the VVL.

#### 3.2.2. Type B2 RH—B1 plus Paracervical Lymphadenectomy

As mentioned above, the lateral part of the paracervix mainly contains lymphatics, which can be considered a lymph node-bearing area. Querleu defined the paracervical lymph nodes as located dorsal to the obturator nerve, ventral to the sciatic nerve roots, and lateral to the ureter [12,23,26]. Gluteal and pudendal lymph nodes, located dorsal to the internal iliac vascular system, are part of the paracervical lymph nodes [26]. Generally, lymph nodes located dorsal to the internal iliac vessels and nodes between the obturator nerve and lumbosacral trunk are removed during paracervical lymphadenectomy. The obturator nerve is the main landmark for distinguishing paracervical lymph nodes from pelvic parietal nodes. The pelvic parietal lymph nodes are located ventrally and laterally to the obturator nerve [14,23,26,30]. Paracervical lymphadenectomy and pelvic parietal lymphadenectomy constitute a comprehensive pelvic lymph node dissection. Importantly, paracervical lymphadenectomy is not associated with injury to the pelvic autonomic nerves, as it avoids clamping the paracervix at the pelvic wall together with the nerves and vessels. Therefore, paracervical dissection enhances lateral radicality without increasing morbidity [12,19,22,23,26,30]. However, there is always a possibility of injury to the deep vessels (the visceral tributary of the internal iliac vein or gluteal veins) and nerves located in the lateral part of the paracervix.

##### Comments

The definition corresponding to the lateral part of the ureter and medial/dorsal to the obturator nerve does not mention the paracervical lymph node area, and it is not enough to clarify the margin. To clarify the margin of the paracervical lymph nodes, the plane lateral to the internal iliac vessel system should be stated. Therefore, the PSNs, which are mainly in the zone of the lateral (distal) paracervix (the PSNs arise in the zone of the lateral pararectal space and run towards the caudal part of the medial pararectal space, located medial to the internal iliac vascular system), should not be injured during paracervical lymphadenectomy.

### 3.3. Type C RH—Classic Radical Hysterectomy

Type C has been precisely described in our previous articles [18,31]. It is divided into two subtypes: Type C1 (nerve-sparing RH) and C2 (classic RH without preservation of the PANS). The main resection line for three supraureteric parametria in Type C RH is at the level of the internal iliac artery laterally, the bladder ventrally, and the rectum dorsally. Nevertheless, the resection line of the three infraureteric parametria depends on the type of RH—C1 or C2.

#### 3.3.1. Type C1 RH—Nerve-Sparing Radical Hysterectomy

Type C1 RH requires the development of four lateral avascular spaces: the medial and lateral paravesical, medial (Okabayashi’s pararectal), and lateral pararectal (Latzko’s pararectal) spaces. Furthermore, two spaces are developed at the ventral parametrium: the ureteral tunnel space (for ureteric dissection at the paracervical zone) and Okabayashi’s paravaginal space (for total lateralization of the distal ureter to the ureterovesical junction) [28,32,41]. Development of Yabuki’s space is not required, as its dissection reveals the parasympathetic vesical branches partly but not the entire topography of vesical nerve branches that have a direct connection with the IHP and bladder base [28]. Yabuki tried to identify the vesical nerve branches in the VVL despite the fact that most of the articles stated that the vesical nerve branches are not part of the VVL [27,28,42]. However, Yabuki’s space has applications during Type C1 RH [18,31] that provide a safe dissection between the VUL and the ureter and a cleavage for the proximal part of the VVL. Querleu et al. described a potential space located near the ureterovesical junction and at the caudal portion of the ventral parametrium as the “paravaginal space”, which was originally described by Okabayashi in 1921 (The limits of Okabayashi’s paravaginal space are as follows: laterally, the distal ureter and VVL; medially, the lateral vaginal wall- paracolpium; ventrally, the VUL; dorsally, the medial part of the paracervix) [22,26,41]. The rectovaginal ligament is resected following the development of Okabayashi’s pararectal space and the lateralization of the ureter, mesoureter, and HN. The IHP is dissected laterally from the rectovaginal ligament so the uterovaginal nerve plexus can easily be resected with the paracervix. The vesical nerve branches could be preserved by developing Okabayashi’s paravaginal space and meticulous dissection of the VVL. The vaginal extent of the tumor determines the length of vaginal resection.

Consequently, during Type C1 RH, the supraureteric parametrium—laterally parauterine, ventrally vesicouterine, and dorsally rectouterine tissue—are totally transected, and the infraureteric parametrium—laterally paracervix and ventrally vesicovaginal—are partially resected. The dorsal part of the infraureteric parametrium (the rectovaginal ligament) is totally transected at the level of the rectum.

##### During Type C1 RH, the Resection Lines of the Three Parametria Are as Follows

1. Ventral parametrium
  - Transverse: The resection line of the VUL is at the level of the bladder.
  - Longitudinal: The resection line is formed by the vesical nerve branches, which are identified dorsolaterally to the course of the distal ureter after the development of Okabayashi’s paravaginal space. The VVL is dissected from the paracervix/paracolpium by preserving the vesical nerve branches, and only the cranial (proximal) part of the ligament is resected.
2. Lateral parametrium
  - Transverse: The resection line of the parauterine and paracervix tissue is at the axis of the internal iliac artery.
  - Longitudinal: The paracervix tissue is resected at the level of the deep uterine vein (vaginal vein), regarding the preservation of the PSNs, which lie dorsal to the deep uterine vein.
3. Dorsal parametrium
  - Transverse: Resection of the rectovaginal ligament is performed at the level of the rectum, considering that the uterosacral (rectouterine) ligament is a peritoneal fold, not a true ligament, which is dissected and resected at the level of the rectum to reach the entire rectovaginal ligament.
  - Longitudinal: The HN and the mesoureter are dissected laterally from the rectovaginal ligament. The proximal part of the IHP is identified (during the dissection of the RVL—dorsolateral to the upper vagina) and spared. The caudal limit of resection of the rectovaginal ligament depends on the resection plane of the vagina.

**Figure 13:**
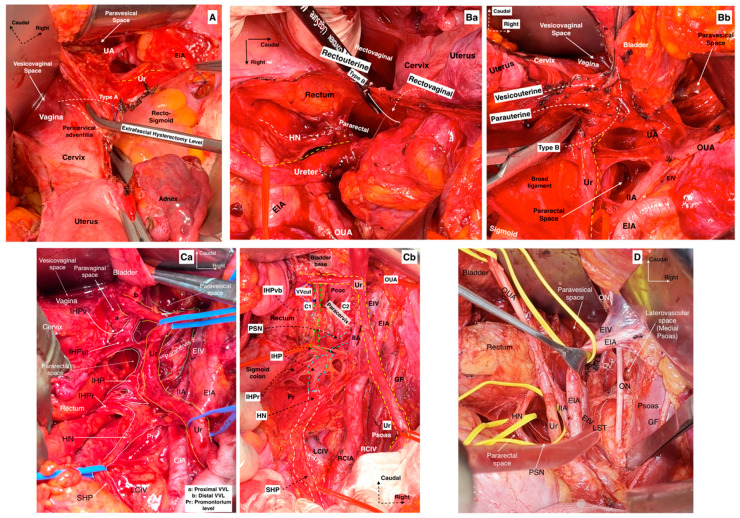
(**A**): Type A minimal RH, showing the lateral parametrium/paracervix resection level between the pericervical adventitia and ureter. (**B**): Type B-modified RH, demonstrating the parametrium resection level according to the longitudinal axis of the ureter. (**Ba**): Dorsal parametrium and (**Bb**): lateral and ventral parametrium. (**Ca**): Pelvic autonomic (hypogastric) nerve plate, ureter, and vesicovaginal ligament. Resection of only the proximal (cranial) part of the vesicovaginal ligament without injuring the inferior hypogastric plexus vesical branches is the critical point of Type C1 RH. (**Cb**): Type C1 resection level at the ventral aspect of the inferior hypogastric plexus vesical branches and the remaining paracervix, which is removed during Type C2 RH without nerve sparing. (**D**): Paracervical lymph node area and pelvic sidewall; dorsal to the obturator nerve, ventral to the sciatic roots/lumbosacral trunk, and lateral to the internal iliac vessels. Developing the laterovascular plane, which is also called the medial psoas plane, located lateral to the external–internal iliac vessel system and medial to the psoas major muscle, facilitates paracervical lymphadenectomy (Open surgery; surgical dissection by author I.S.). CIA: common iliac artery, LCIV: left common iliac vein, EIV: external iliac vein, EIA: external iliac artery, IIA: internal iliac artery, OUA: obliterated umbilical artery, Ur: ureter, GF: genitofemoral nerve, RCIA: right common iliac artery, RCIV: right common iliac vein, Pr: promontorium, VVL: vesicovaginal ligament, VV: vesicovaginal ligament, SHP: superior hypogastric plexus, HN: hypogastric nerve, PSN: pelvic splanchnic nerves, IHP: inferior hypogastric plexus, IHPv(b): inferior hypogastric plexus vesical branches, IHPut: inferior hypogastric plexus uterine branches, IHPr: inferior hypogastric plexus rectal branches, UA: uterine artery, LST: lumbosacral trunk, ON: obturator nerve, OV: obturator vein.

Some aspects of the resection lines are shown in Figure 13.

##### Comments

After resection of the rectouterine and rectovaginal ligament, parauterine tissue, and part of the paracervix with the VUL, the ureter is completely unroofed from the dorsal and lateral parametrium and partially from the ventral parametria. A key point of the Type C1 RH is developing the paravaginal space so that the VVL can be identified and partially resected without damaging the vesical nerve branches of the IHP. In this area, the paravaginal veins (vesicovaginal venous plexus), which drain into the vaginal vein, can be injured, and that will cause problematic bleeding (Figure 6). During Type C1 RH, the dorsal part of the paracervix and the caudal (distal) part of the VVL are not excised. For Type C1 RH, the paracervical lymphadenectomy was not mentioned in the original document [14,26]. However, paracervical lymphadenectomy should be an integral part of Type C1 RH in addition to pelvic parietal lymphadenectomy, as comprehensive pelvic lymphadenectomy.

#### 3.3.2. Type C2 RH—Classic Radical Hysterectomy

Type C2 RH entails complete parametrial resection. The ureter is fully mobilized from both the VUL and the VVL up to the bladder wall. Okabayashi’s pararectal and paravaginal spaces are totally dissected. The development of Okabayashi’s paravaginal space provides safe lateralization of the ureter. It is not used to isolate and spare the vesical nerve branches. The pelvic autonomic nerves are part of the postoperative specimen, as they are excised and sacrificed [15,23]. The resection length of the vagina is approximately >20 mm but depends on the vaginal or pericervical extent of the disease. However, for FIGO stage IIA CC, where the vaginal fornices are involved by the tumor, more extensive vaginal and paracolpium resections are required [12,26]. Querleu et al. mentioned that this type of surgery should be performed only for surgical anatomical reasons for bulky or high-risk tumors, whereas Type C1 is the preferable procedure [23].

During Type C2 RH, the resection lines of the three parametria are as follows [14,15,23]:

1. Ventral parametrium
  - Transverse: Complete resection of the VUL at the level of the bladder and total dissection and lateralization of the distal ureter up to the ureterovesical junction.
  - Longitudinal: The resection line depends on the level of the paracolpium and vaginal cuff resection, primarily to the level of the pelvic floor (the levator ani/pubococcygeus muscle), transecting the entire VVL, some parts of the paracolpium, and the vesical nerve branches of the IHP.
2. Lateral parametrium
  - Transverse: The lateral resection line is at the axis of the internal iliac artery.
  - Longitudinal: Complete resection of the LP. The paracervix and parts of the paracolpium are entirely removed, extending primarily to the level of the pelvic floor (the levator ani/iliococcygeus muscle). Thus, the paravesical and pararectal spaces merge into one entity. The PSNs, which are located at the dorsal part of the paracervix, are transected.
3. Dorsal parametrium
  - Transverse: Resection of the rectovaginal ligament at the level of the rectum.
  - Longitudinal: Maximal dorsal resection of the rectovaginal ligament, deep to the sacral fascia attachments, sacrificing the HN and part of the PSNs along with the IHP.

Some aspects of the resection lines are shown in Figure 13.

##### Comments

Class III Piver–Rutledge–Smith RH or type III RH in the EORTC (Mota) classification includes excessive resection of the vagina, irrespective of the tumor’s localization, involving the removal of the upper third of the vagina [11,13]. Class III Piver–Rutledge–Smith RH emphasizes the preservation of the superior vesical artery and avoidance of ureteral dissection at the level of the bladder where the artery is situated [13]. The superior vesical artery is a useful landmark for identifying the visceral tissue of the bladder. During Type C2 RH, total ureteric lateralization of the upper vagina by developing Okabayashi’s paravaginal space is critical to reaching and excising all the paracervix (Figure 7b). Although the paracervical lymph nodes are mostly resected as a component of the resection margins during Type C2 RH, it will be better to state paracervical lymphadenectomy in the explanation of Type C2 RH as a part of the comprehensive pelvic lymphadenectomy. We also believe that the longitudinal resection line of the lateral parametrium should be at the level of the internal iliac artery, not at the level of the internal iliac vascular system, as stated by Cibula et al. [15] and Querleu et al. [23]. The internal iliac vein is a variable vein with different draining patterns and should not be used as a landmark for determining radicality.

#### 3.3.3. Selective Systematic Nerve–Sparing Type C2 Radical Hysterectomy (Type C2N)

Currently, different authors in Europe have adopted the Japanese nerve-sparing concept of RH [12,18,31,41,42,43]. The entire IHP (without the uterovaginal nerve plexus), together with the vesical nerve branches, is clearly visualized and preserved. The authors have shown that the entire VVL with the paracervix and paracolpium can be resected, and the vesical nerve branches can be spared [12,18,31,41,42,43].

##### Comments

The nerve-sparing approach can be divided into two subtypes: Type C1, which is total nerve-sparing and Type C2, which is selective systematic nerve-sparing and preferred to state as ‘C2N’. The difference between these two subtypes is the resection margin of the VVL and paracervix.

In Type C1 total nerve-sparing RH, the proximal part of the VVL is excised together with the medial and ventral part of the paracervix, which corresponds to the medial parts of the deep uterine (vaginal) vein, lying on the medial aspect of the pelvic nerve plate. The proximal VVL is meticulously dissected at the ventral part of the vesical nerve branches, so the vesical nerve branches of the IHP are totally preserved.

Type C2 Selective-Systematic Nerve-Sparing RH maintains resection of the entire paracervix along with paracervical lymph nodes without sacrificing the pelvic autonomic nerves. In Type C2 Selective-Systematic Nerve-Sparing RH, the VVL is totally resected from the level of the bladder base with the paracolpium (the medial component of the paracervix up to the level of mid-vagina). During this step, the vesical nerve branches are selectively dissected and lateralized from the paracolpium and, therefore, preserved. The deep uterine (vaginal) vein and the paracervix are totally resected along with components of the vesicovaginal venous plexus. During this step, the PSNs are selectively dissected from the distal/dorsal part of the paracervix and preserved. The rectouterine ligament is totally dissected after lateralization of the HN, and the rectovaginal ligament is totally resected after lateralization of the IHP.

**Figure 14:**
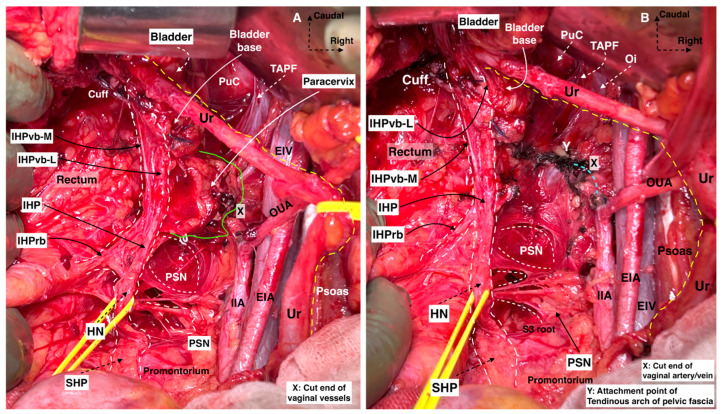
Selective-Systematic Nerve-Sparing Radical Hysterectomy. (**A**): First step: resection of the parametrium ventromedial to the pelvic hypogastric nerve plate (rectouterine/rectovaginal, parauterine/medial paracervix, vesicouterine, and vesicovaginal). (**B**): Second step: resection of the distal paracervix and paracervical lymph nodes lateral to the pelvic hypogastric nerve plate. The tendinous arch of pelvic fascia or ischial spine level is the distal landmark of paracervix resection (Open surgery; surgical dissection by author I.S.). EIV: external iliac vein, EIA: external iliac artery, IIA: internal iliac artery, OUA: obliterated umbilical artery, Ur: ureter, SHP: superior hypogastric plexus, HN: hypogastric nerve, PSN: pelvic splanchnic nerves, IHP: inferior hypogastric plexus, IHPvb: inferior hypogastric plexus vesical branches, M: medial, L: lateral, IHPrb: inferior hypogastric plexus rectal branches, PuC: pubococcygeus, TAPF: tendinous arch of pelvic fascia, Oi: obturator internus muscle, S: sacral.

**Figure 15:**
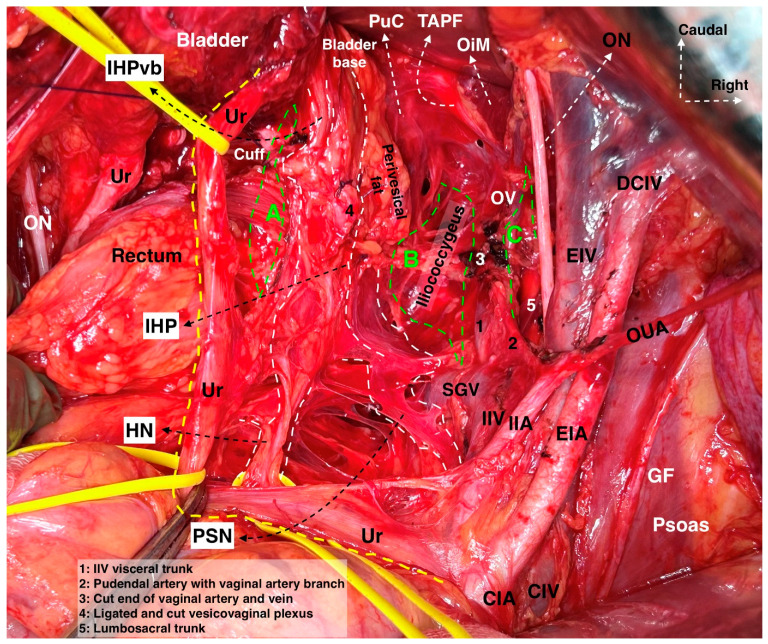
Three-step surgery for Selective-Systematic Nerve-Sparing Radical Hysterectomy. The anatomy after surgery reveals that all the lateral, ventral, and dorsal parametria are resected. A—ventromedial plane to pelvic hypogastric nerve plate. B—lateral plane to pelvic hypogastric nerve plate. C—dorsolateral plane to the obturator nerve and internal iliac vessels. Indeed, the surgery can be performed in one step: an en-bloc resection of the three parametria, selectively sparing the pelvic hypogastric nerve plate by meticulous dissection of the parametria (Open surgery; surgical dissection by author I.S.). CIA: common iliac artery, CIV: common iliac vein, EIV: external iliac vein, EIA: external iliac artery, IIA: internal iliac artery, OUA: obliterated umbilical artery, DCIV: deep circumflex iliac vein, IIV: internal iliac vein, SGV: superior gluteal vein, Ur: ureter, GF: genitofemoral nerve, OV: obturator vein, ON: obturator nerve, PuC: pubococcygeus, TAPF: tendinous arch of pelvic fascia, OiM: obturator internus muscle, HN: hypogastric nerve, PSN: pelvic splanchnic nerves, IHP: inferior hypogastric plexus, IHPvb: inferior hypogastric plexus vesical branches.

Type C2 Selective-Systematic Nerve-Sparing RH can be performed in three steps based on the axis of the pelvic hypogastric nerve plate. First, the parametria (rectouterine/rectovaginal, parauterine/medial paracervix, vesicouterine, and vesicovaginal) ventromedial to the pelvic hypogastric nerve plate are resected with an appropriate vaginal margin (Figure 14). Second, the distal paracervix, lateral to the pelvic hypogastric nerve plate, is resected. Finally, the paracervical lymph nodes, located dorsal to the obturator nerve, are resected (Figure 15).

### 3.4. Type D—Laterally Extended Resection for RH

Type D RH includes rare ultraradical procedures (super RH), mainly indicated for locally advanced, radio-chemotherapy-resistant cervical tumors or pelvic sidewall recurrences [14,15,23]. The main resection margin for Type D RH is the pelvic sidewall.

#### 3.4.1. Type D1 RH—Laterally Extended Parametrectomy (LEP)

Type D1 RH aims for the resection of the entire paracervix along with the hypogastric vessels (artery or vein, gluteal–internal pudendal–obturator branches) from the pelvic sidewall line. Palfalvi and Ungar first described the laterally extended parametrectomy (LEP), which corresponds to Type D1 RH [44,45]. The procedure consists of the removal of the entire paracervix together, not only with the visceral but also with parietal vessels of the internal iliac system (inferior and superior gluteal, iliolumbar, internal pudendal, and obturator vessels) in order to expose the ventral roots of the sciatic nerve [23,44,45]. Consequently, the internal iliac artery and vein are cut and divided, and no connective and lymphatic tissue remains on the pelvic sidewall. Therefore, the main purpose of LEP is to remove the entire paracervix together with lymph nodes located dorsal and lateral to the internal iliac vessels (gluteal and pudendal lymph nodes, which are not routinely removed during classical RH) by extending the lateral boundaries of dissection to the true pelvic sidewall. The procedure is indicated for selected IB3 CC cases, stage IB CC with lymph node metastases, and stage IIB CC [44,45].

##### Comments

Mibayashi introduced a similar extended procedure in Japan in 1941—“supra-radical hysterectomy”, which consisted of extensive dissection of the cardinal ligament (paracervix) and resection of the entire internal iliac vessel system [46]. Nowadays, it seems that the LEP procedure has no applications, as the majority of indications for the procedure are actually contraindications for any type of RH in CC patients. Firstly, most of the mentioned lymph nodes are removed during paracervical lymphadenectomy without resection of the vessels and nerves. Secondly, the initial indications for LEP are debatable and not part of most guidelines [6,47]. Currently, the ESGO guidelines for managing patients with CC do not include LEP in the template, as the mentioned indications are nowadays considered suitable for definitive radiotherapy or chemoradiotherapy [6,47]. Nevertheless, we believe that LEP should be maintained as a surgical procedure, which could be initiated for patients with resistant or recurrent CC after definitive radiotherapy or chemoradiation. In some cases of persistent or recurrent CC tumors, LEP could achieve clear resection margins. LEP is associated with reduced morbidity compared to laterally extended endopelvic resection, as it is a less radical procedure [44,45,48]. However, a multidisciplinary team must determine which type of procedure should be performed in CC recurrence cases—LEP or laterally extended endopelvic resection in order to obtain negative resection margins. LEP after radiotherapy should be performed by trained and skilled surgeons, as dissection of avascular space and the three parametria of the uterus is challenging. It is imperative to mention that the surgical technique for the procedure (as part of the ultra-radical type of hysterectomy) must be passed on to future generations of young surgeons, as every oncogynecologist has to be able to perform it.

#### 3.4.2. Type D2 RH—Laterally Extended Endopelvic Resection (LEER)

Type D2 RH includes D1 plus resection of adjacent fascial and muscular structures (obturator fascia and obturator internus muscle ventrally, the coccygeus muscle dorsally, and the sacrospinous ligament dorsolaterally). The procedure corresponds to laterally extended endopelvic resection (LEER). This procedure is divided into three types—anterior, total, and posterior. LEER procedures are part of cancer field theory, so resection of other visceral organs in the pelvis (rectum, bladder) are included in the template, as they are part of the compartmental surgery. Anterior LEER is indicated for ventrally invasive ontogenetic stages and includes resection of the uterus together with the bladder. Total (anteroposterior) LEER is indicated for ventrally and dorsally invasive ontogenetic stages and represents the removal of the uterus, bladder, and rectum. The posterior LEER is performed rarely, and it includes the removal of the uterus with the rectum. Indications for different types of LEER depend mainly on the different types of pelvic sidewall recurrences. Some parts of the oT4 cancer field (involvement of the bladder mucosa, anterior rectal wall) could be a part of total LEER. However, generally, the oT4 ontogenetic stage is a contraindication for LEER, as complete removal is hardly achievable due to the metastatic involvement of most tissues of the lower trunk. Moreover, the prognosis is not favorable despite RO resection [48,49,50,51]. Therefore, contraindications for LEER are metastatic involvement of the following: psoas major muscle, external iliac vessels, bones, sciatic nerve/foramen, and sacral roots. These cases are in the oT4 ontogenetic stage, and surgery is not an option [48,49,50,51].

There are three pathomechanisms of pelvic sidewall tumor fixation—to fascia, to the internal iliac vessels, and to the lymph nodes. Usually, lateral tumor fixation on the pelvis is due to a desmoplastic reaction on muscle aponeurosis (not direct invasion), but the muscles are resected to secure a clear margin. Anatomical structures (besides organs) that could be resected during LEER are the following: the muscles (pubococcygeus, iliococcygeus, coccygeus, and obturator internus muscles) and the internal iliac vessels. However, any sign of muscle tumor involvement in imaging modalities is a contraindication for LEER. As mentioned above, the muscles are resected just to achieve RO resection. Similarly, the tumor-invaded urogenital mesentery is resected up to its origin [48,49,50,51].

##### Comments

**Figure 16:**
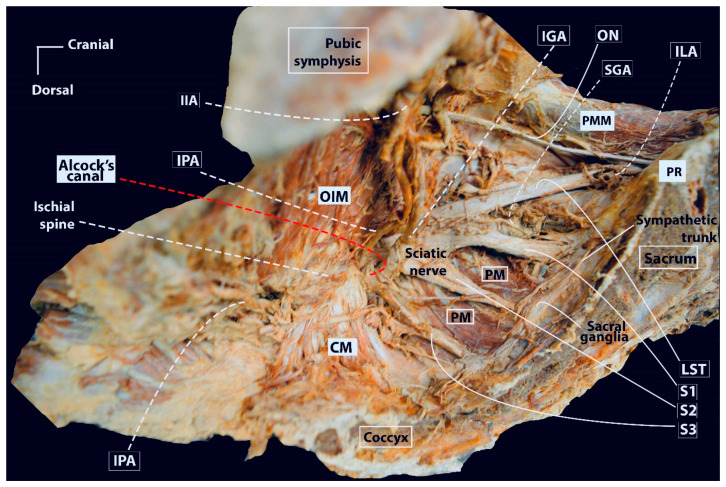
Pelvic sidewall anatomy (embalmed cadaver—dissection by author S.K.). The posterior branch of the internal iliac artery is cut. The artery is elevated ventrally in order to identify the sacral plexus. IIA—internal iliac artery; IPA—internal pudendal artery; OIM—obturator internus muscle; CM—coccygeus muscle; PM—piriformis muscle; IGA—inferior gluteal artery; ON—obturator nerve; SGA—superior gluteal artery; ILA—iliolumbar artery; PMM—psoas major muscle. PR—promontory; LST—lumbosacral trunk; S1, S2, and S3—ventral rami of the sacral plexus.

In the update of the Q–M classification, it is stated that Type D2 RH includes D1 plus resection of the obturator fascia/muscle, the coccygeus muscle, the pelvic part of the piriformis muscle, the sacrospinous ligament, and acetabulum [23,49,50,51]. However, resection of the piriformis muscle is not a part of the surgical steps of LEER, according to the articles by Höckel [48,49,50,51]. It is quite logical, as the muscle is located dorsal to the sacral roots and the sciatic nerve (Figure 16). Moreover, in the Q–M classification system, it is mentioned that the removal of other organs is not included in the template, and Type D2 RH is highlighted as a “procedure, which corresponds to LEER as described by Höckel” [14,23]. Therefore, the term “modified LEER—without resection of other organs” can be used instead; as seen above, LEER is always associated with bladder and/or rectal resection. Additionally, the sacrospinous ligament lies dorsal (inferior) to the levator ani, and the acetabulum resection is far from the main aim of the RH. Therefore, Type D2 is an exenterative procedure and should be removed from the RH classification and replaced with the term “modified LEER” with no resection of other organs.

Nowadays, LEER is rarely performed, as its indications are limited due to advancements in irradiation techniques. However, it still has applications for the surgical management of patients with pelvic sidewall recurrences [6].

Type IV or Class IV RH in other classifications does not include the removal of branches of the internal iliac vessels or muscle and fascia. However, up to three-quarters of the vagina is transected [11,13]. In turn, the Piver–Rutledge–Smith and the EORTC classifications include an additional Class V or Type V, which represents partial removal of organs adjacent to the uterus (ureters, bladder, rectum). The Q–M classification does not include procedures associated with the removal of other organs or structures. This is quite logical, as it corresponds to stage IV of the disease—an indication for primary chemoradiation [15,23].

**Figure 17:**
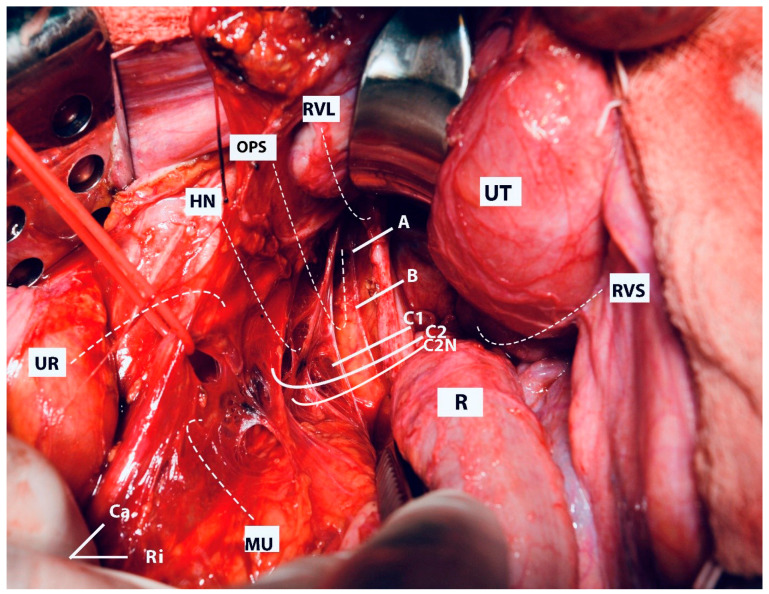
Resection lines of the rectovaginal ligament during different types (from A to C2N) of radical hysterectomy (left pelvic sidewall) (Open surgery; surgical dissection by authors Y.K. and S.K.). RVL—rectovaginal ligament; OPS—Okabayashi’s pararectal space; HN—hypogastric nerve; UR—ureter; MU—mesoureter; R—rectum; RVS—rectovaginal space; UT—uterus; Ca—caudal; Ri—right.

**Figure 18:**
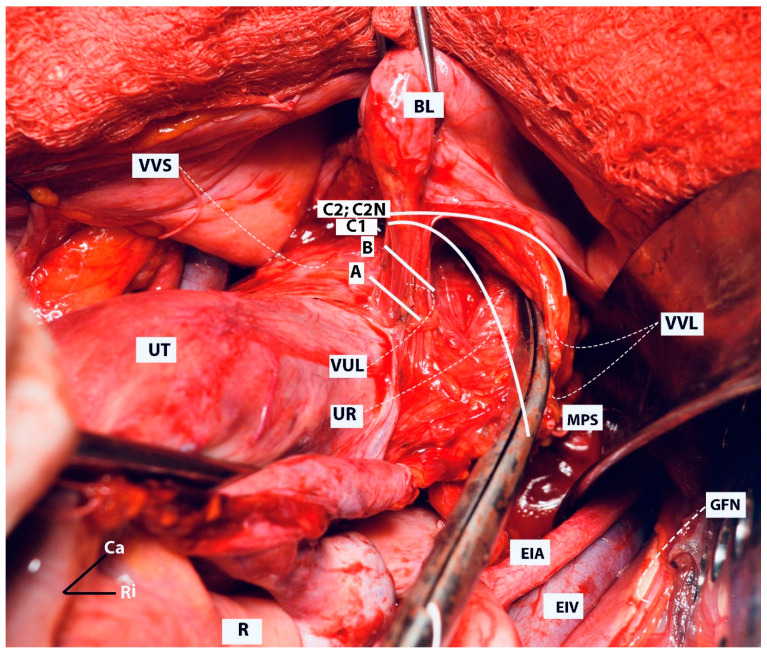
Resection lines of the ventral parametria during different types (from A to C2N) of radical hysterectomy (Open surgery; surgical dissection by authors Y.K. and S.K.). MPS—medial paravesical space; VVS—vesicovaginal space; UT—uterus; R—rectum; VUL—vesicouterine ligament; VVL—vesicovaginal ligament; EIA—external iliac artery; EIV—external iliac vein; GFN—genitofemoral nerve; UR—ureter; C1—partial nerve-sparing; C2N—selective-systematic nerve sparing; Ca—caudal; Ri—right.

Figure 17 and Figure 18 show the resection lines of the ventral and dorsal parametria for the described types of RH.

## 4. Discussion

Lührs et al. conducted a prospective study investigating the incidence of lymph nodes and lymph node metastases in the upper paracervical lymphovascular tissue (later defined by Querleu as the parauterine lymphovascular tissue) among 145 women with early-stage cervical cancer (most cases were stages IA1–IB1 according to the FIGO classification 2009). Surgeries and identification of the sentinel node were predominantly conducted using a robotic-assisted approach. At least one parauterine lymph node was detected in more than half of the patients (52.4%), indicating that the parauterine lymphatic channel is one of the main potential lymph pathways in early-stage CC. Pelvic lymph node metastases were identified in 19 women (13.1%). Metastatic parauterine lymph nodes were detected in six women (2.1% of all patients, 31.5% of those with positive nodes), of which three patients (15.8% of those with positive lymph nodes) had only isolated parauterine lymph nodes without the presence of positive pelvic lymph nodes. The metastatic involvement of parauterine lymphatic tissue in early CC cases was supported by the fact that the median tumor size in patients with parauterine lymphatic metastases was 12–30 mm. It should be stressed that all patients with parauterine lymphatic metastases had positive lymphovascular space invasion in the final histology [29].

Lührs et al. questioned whether the dissection of PALT through open surgery could be applicable [29]. However, Querleu later stated that PALT is generally removed during the transection of the uterine artery (along with the superficial uterine vein) at its origin from the internal iliac artery, which corresponds with the resection of the parauterine tissue [30]. Removal of the PALT is not associated with pelvic autonomic nerve injury and subsequent morbidity [29,30]. Therefore, the authors concluded that PALT removal, even without total transection of the uterine artery, should be an integral part of every hysterectomy for CC, as it allows the removal of so far neglected lymphatics [29,30]. That should be critically mentioned for Type A RH, and the resection of the PALT can be added to Type A RH to excise possible lymphatic metastatic areas along the parauterine pathway, where the paracervix is minimally resected. Type A RH requires training in gynecologic oncology and should be performed in high-volume gynecologic oncology hospitals. Understanding the surgical concept of Type A minimal RH is mandatory for future clinical trials that will compare the overall survival of less radical surgery versus radical surgery. Unfortunately, the recently published SHAPE trial did not consider the Q–M classification and compared the oncologic outcomes (primarily pelvic recurrence) between simple and radical hysterectomy (Type II) in patients with low-risk early-stage CC (tumor diameter of 2 cm or less with limited (<50%) stromal invasion) [40].

**Figure 19:**
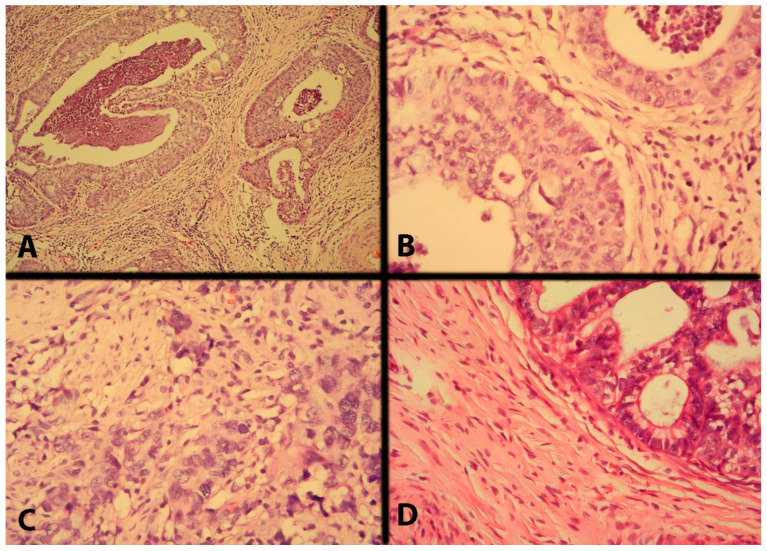
The three histological tumor growth patterns of cervical cancer—the comedo-like ((**A**)—10× H&E, (**B**)—40× H&E), the infiltrative ((**C**)—40× H&E), and the expansive ((**D**)—40× H&E) (histological evaluation and diagnosis by author I.I.). (**A**)—pseudocomedo pattern demonstrating comedo-like necrosis in the center of tumor nests with smooth outlines, resembling glandular structures colonized by in situ adenocarcinoma of the uterine cervix. (**B**)—pseudocomedo growth pattern of cervical adenocarcinoma demonstrating debris-filled clefts surrounded by several layers of cancer cells. (**C**)—Infiltrative growth pattern of squamous cervical cancer demonstrating groups of cancer cells infiltrating the surrounding stroma in a chaotic manner. (**D**)—expansive growth pattern in adenocarcinoma of the uterine cervix, demonstrating cribriform growth and pushing margin interference with the surrounding stroma, closely mimicking a benign lesion.

Additionally, one of the most significant concepts in modern cervical cancer surgery is the concept of tailoring surgical radicality. That means that oncogynecologists should avoid the theory of “one-fits-all” during cervical cancer surgery. Type A RH may not be recommended for selected patients with lymphovascular space invasion or deep stromal invasion and for the identification of novel predictive factors to modulate the surgical radicality, such as the currently mentioned comedo-like growth pattern in invasive early-stage cervical cancer (Figure 19). Cosma et al. investigated 87 women with squamous cervical cancer, the majority of which were classified as pathological FIGO stage I. The authors found three histological tumor growth patterns of cervical cancer: comedo-like, infiltrative, and expansive. The authors found that the comedo-like growth pattern was associated with an increased risk of parametrial involvement and lymph node metastases. Therefore, investigating the tumor growth patterns in a conization specimen before a hysterectomy may be useful for tailoring surgical radicality [52].

**Figure 20:**
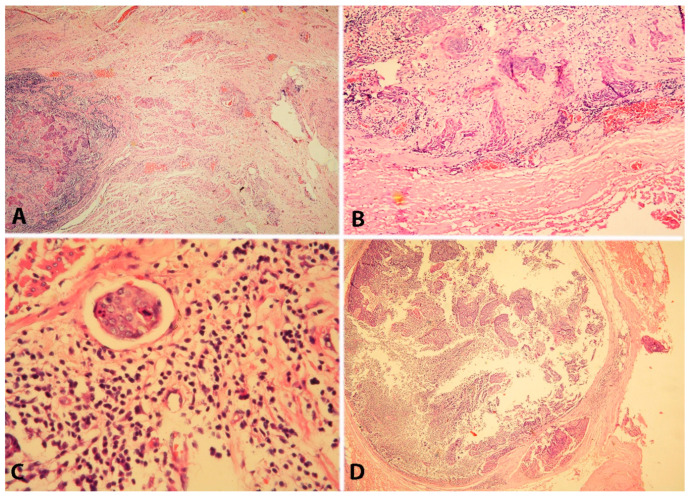
Different types of parametrial involvement of the paracervix in cases of cervical cancer (histological evaluation and diagnosis by author I.I.). (**A**)—continuous involvement 4× H&E; (**B**)—discontinuous 10× H&E; (**C**)—metastases to lymphatic channel 40× H&E; (**D**)—parametrial lymph node metastases 4× H&E.

Parametrial involvement due to cervical tumors is often divided into four types: I—continuous (direct extension of the primary tumor), II—discontinuous (cancer cells identified in the parametrium, without any relation to specific anatomical structures), III—metastases to the lymphatic vessels, and IV—involvement of the parametrial lymph nodes [53,54,55]. Four types of parametrial involvement are shown in Figure 20.

Some authors have simplified parametrial involvement into continuous and discontinuous patterns, with the latter including both lymphatic vessel involvement and parietal lymph node involvement [56]. Historically, the significance of parametrial lymph nodes has been highlighted. Burghardt et al. [53] and Girardi et al. [54], in their examinations of lateral parametrium lymphatics in giant sections of 359 surgical specimens from women with stages IB, IIA, and IIB, found the presence of parametrial lymph nodes in 78% of the specimens, of which 22.5% showed parametrial metastatic involvement. Among patients with positive parametrial lymph nodes, 44.4% were found in the medial part of the lateral parametrium near the cervix, 38.1% in the lateral part, distal paracervix, and the pelvic sidewall, and 17.5% in both parts. The authors observed all four types of parametrial involvement but noted that continuous spread of the tumor is rare. In stage IB1 tumors, the most common types of parametrial involvement were Types IV (11.4%) and III (3%). The incidence of positive parametrial lymph nodes correlated with the stage of the disease (11.4% for stage IB and 21.5% for stage IIB) and tumor volume, indicating that the status of the parametrial lymph nodes is a reliable indicator of pelvic lymph node status. Interestingly, parametrial lymph nodes were positive in 3.4% of patients with the smallest tumor. The authors concluded that the extent of parametrial resection should be close to the pelvic sidewall in order to remove possible positive parametrial lymph nodes [53,54]. Girardi et al. observed recurrences in 31% of the patients, of which 32.5% had positive parametrial lymph nodes, whereas positive parametrial lymph nodes were found in 10.6% of patients who were free of recurrence. The 5-year survival rate was lower in the group with metastatic parametrial nodes [54].

Winter et al. investigated parametrial lymph nodes among 351 women (FIGO stages IB, IIA, and IIB) with negative pelvic lymph nodes. All patients underwent Piver–Rutledge–Smith Class III RH and pelvic lymphadenectomy. Overall, 44 (12.5%) of the women had parametrial metastatic involvement. The incidence of continuous parametrial involvement increased with tumor volume, whereas the frequency of discontinuous, lymphatic vessel, and lymph node involvement was irrespective of tumor stage or size. The incidence of isolated medial and lateral parametrial involvement was 3.8% and 2.2%, respectively, among 180 patients with small tumors (less than 2.1 cm). Thus, Type B1 modified RH for stage IB1 tumors, with resection only of the medial paracervix, would presumably have left possible tumor metastasis to the lateral paracervix intact. This data suggests that discontinuous parametrial involvement could be a reason for recurrent disease [55]. Winter et al. found that parametrial involvement had no impact on disease-free survival among women with negative pelvic lymph nodes who underwent Type III radical hysterectomy with pelvic lymph node dissection [55]. However, Type B2-modified RH would definitely leave an in situ tumor in the lateral paracervix. Most patients in Winter et al.’s study was considered at high risk of recurrence and received chemotherapy or radiotherapy. A combined treatment option (surgery followed by radiotherapy/chemoradiotherapy) is not recommended by the current guidelines for the surgical treatment of CC [6,47]. Puente et al. conducted a similar study (CC patients with IB1 and IIA) and reported a 2.3% incidence of positive lateral paracervical lymph nodes with negative pelvic lymph nodes [57]. In contrast, Querleu et al. reported no paracervical lymph node metastases among 38 patients with small tumors (less than 2 cm in diameter). However, they noted that paracervical lymphadenectomy could prevent long-term latero-pelvic recurrences, and it is not time-consuming or associated with additional postoperative morbidity [58]. In two studies, Panici et al. found a high presence of metastases in the three parametria, specifically at the parauterine tissue and vesicouterine/vesicovaginal tissue, in hysterectomy specimens from radical surgeries for CC stages IB1–IIA1 [59,60]. Höckel et al. also stated that the perispinous adipose tissue, which contains the pelvic plexus, the PSNs, small blood vessels, and lymphatic tissue, is not removed during Type III RH (Piver–Rutledge–Smith classification) and is a frequent site of tumor recurrence [61]. It should be stressed that all the mentioned studies used the old FIGO classification for CC [62]. All these studies show that paracervical lymphadenectomy should be an integral part of not only Type B2 RH but also Type C1/C2 RH in order to obtain comprehensive pelvic lymphadenectomy. Additionally, one might consider that even in Type B2 RH, the paracervical lymph nodes are resected, but the lateral paracervix is not transected.

Although the Type B2 RH classification is widely adopted across the world, it is not part of the Korean Gynecologic Oncology Group classification of RH. Korean authors have stated that the differentiation between pelvic and paracervical lymph nodes is not always applicable, and the clinical significance of differentiating between the two Type B subtypes is minimal [63]. This approach is not represented in any other classification of RH [10,11,12,13].

Type C1 RH differs from the Japanese concept of nerve-sparing RH. Several Japanese authors recommend complete dissection of the vesical nerve branches from the VVL; therefore, the VVL is entirely resected at the level of the bladder [24,32,41,64,65,66]. This technical difference can be explained using the difference regarding the extent of vagina/paracolpium resection. Initially, Kobayashi managed to spare the pelvic autonomic nerves, however, without a clear demonstration of the surgical method for preserving the vesical nerve branches [64]. Sakuragi further categorized Kobayashi’s RH into systematic and partial nerve-sparing approaches, where the PSNs and partial portions of the IHP are spared [64,66]. Since then, many authors from Japan have demonstrated surgical techniques for the safe dissection of the vesical nerve branches [24,32,64,65]. In Europe, Muallem et al. introduced nerve-sparing RH even for bulky tumors (IB3 FIGO stage), proposing two types of nerve-sparing RH: Type III (radical hysterectomy with Extended Vaginal-Cuff-Resection) and Type IV (radical hysterectomy with Radical Upper Colpectomy). Type III is equivalent to Type C1 (Q–M) but allows for resection of a longer vaginal vault of approximately 2–4 cm. Type IV is a more radical nerve-sparing RH, indicated for cases of macroscopic vaginal infiltration or IB3 tumors with ventral localization. Contraindications for Type IV Muallem RH (nerve-sparing) include continuous involvement of the paracolpium/paracervix and tumor spread to the tendinous arch of pelvic fascia. In terms of radicality, Type IV is similar to Type C2 but preserves the autonomic nerves [12]. However, the possible presence of perineural invasion in bulky cervical tumors should be considered to ensure oncological safety [67]. Notably, nerve-sparing RH was not included in the Piver–Rutledge–Smith or the EORTC (Mota) classifications [11,13].

Type C2 Selective-Systematic Nerve-Sparing RH maintains resection of the entire VVL and paracervix, including the paracervical lymph nodes without sacrificing the pelvic autonomic nerves, which can be applied to high-risk early-stage CC patients (tumor diameter 3–4 cm plus stromal invasion > 1/2 or with positive lymphovascular space invasion). The understanding of precise pelvic anatomy and proper application of dissection techniques were improved over the years, which led to an anatomical dissection-based surgery for radical hysterectomy, which has decreased surgical morbidity. Moreover, the image-guided radiotherapy techniques were improved and that decreased the radiotherapy-associated morbidity. Consequently, despite the lack of data, Type C2 Selective-Systematic Nerve-Sparing Radical Hysterectomy and image-guided radiotherapy may be acceptable treatments for high-risk early-stage cervical cancer patients if the pelvic lymph nodes are tumor-negative.

It is imperative to mention the contraindications for RH in CC surgery.

According to the latest ESGO/ESTRO/ESP guidelines for CC, RH should be avoided in cases when metastatic lymph node involvement (pelvic or paraaortic) is detected intraoperatively [6]. Completion of RH does not improve survival in patients with intraoperatively detected metastatic lymph nodes, regardless of tumor size or histological type. Such patients should be considered for definitive radiotherapy or chemoradiation [68]. Additionally, patients with preoperatively diagnosed parametrial involvement are not candidates for surgery, as combined treatment (surgery plus radiotherapy) should be avoided in order to reduce morbidity and mortality [47]. Moreover, RH for women with stage T1a1 disease represents overtreatment [6].

It should be stated that the RH classification is not entirely specific to CC. For example, en bloc removal may be indicated for vaginal cancer if the upper vagina is involved by the tumor and for endometrial cancer with continuous parametrial involvement or upper vagina involvement just to get a tumor-free margin. However, the impact of overall survival in endometrial cancer, depending on the radicality of hysterectomy, is controversial [69].

**Figure 21:**
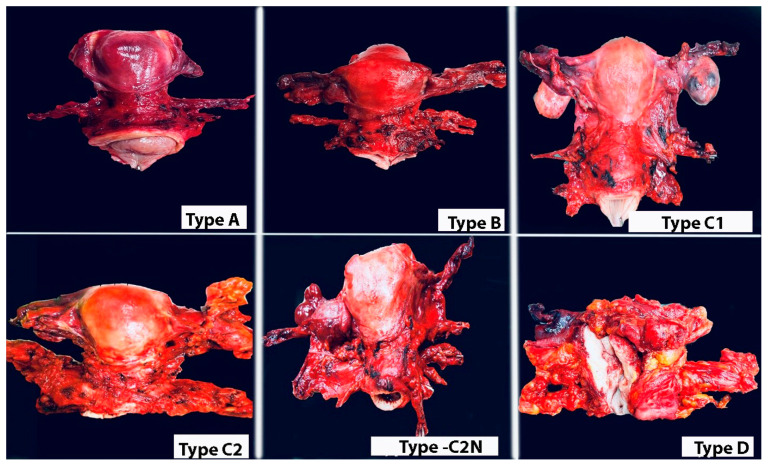
Surgical postoperative specimens of new types of RH according to revised Querleu–Morrow classification (Open surgeries; surgical procedures performed by authors Y.K. and S.K.). All of the ventral or dorsal parts of the three parametria were marked intraoperatively (with clips and different colored stitches), as in the final specimen, it is hard to accurately define and distinguish these anatomical structures. Parauterine lymphovascular tissue, together with the uterine artery/superficial uterine vein, is clearly visible in Types A, B, and C1. In Type C1, the vesicovaginal ligament is partially resected. Type C2—classical radical hysterectomy with total resection of the three parametria together with the majority of pelvic autonomic nerves. Type C2N (Selective-Systematic Nerve-Sparing radical hysterectomy)—the vesicovaginal ligament and paracervix are totally resected. Type D—laterally extended parametrectomy or modified laterally extended endopelvic resection without resection of other organs or anatomical structures. Laterally extended endopelvic resection (Type D2 RH) is shown in Figure Type D.

**Figure 22:**
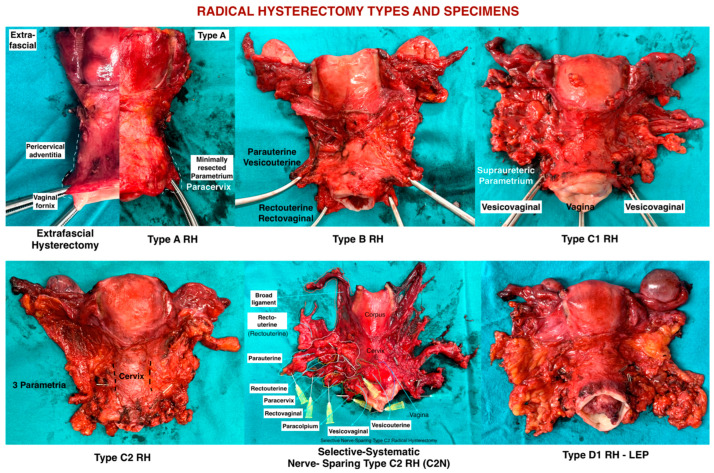
Radical hysterectomy types and specimens. (Open surgeries; surgical procedures performed by author I.S.). Type A RH is minimal resection of the parametrium, and the paracervix is resected medial to the ureter. Type B RH is mainly resection of the parametria at the level of the ureter. Type C1 RH is resection of the proximal VVL with the entire supraureteric ventral, lateral, and dorsal parametria. Type C2 RH is the resection of all supraureteric and infraureteric parametria to the level of the pelvic floor. Type C2N RH is the resection of the entire VVL with the paracervix while selectively preserving the hypogastric nerve pathway. Type D includes laterally extended parametrectomy and modified laterally extended endopelvic resection. Type D1 RH—Laterally extended parametrectomy with resection of the lateral parametria at the pelvic sidewall with the corresponding internal iliac vascular system.

Postoperative specimens after different RH types are shown in Figure 21 and Figure 22. Figure 21 includes the removal of the entire parauterine tissue (PALT together with the uterine artery/superficial uterine vein) during Types A and B RH (the uterine artery is ligated at the level of the internal iliac artery). Figure 22 includes removal of the PALT (without resection of the uterine artery/superficial uterine vein at the level of the internal iliac vessels) in Type A (the uterine artery/superficial uterine vein are transected medial to the ureter) and Type B RH (the uterine artery/superficial uterine vein are transected at the level of the ureter).

Table 1 shows the difference between the types of RH according to the Q–M classification and our update.

## 5. Surgico-Anatomical Tips to Perform Radical Hysterectomy

The pelvic avascular spaces should be developed before starting the resection of the parametrial tissue in lateral, ventral, and dorsal directions. The following steps are performed more or less according to the radicality of the procedure while respecting the anatomical resection margins. The radicality of parametrial resection can be noticed in the surgical specimen (Figure 21 and Figure 22).

The lateral pelvic peritoneum is cut, and the paravesical and pararectal spaces are developed. The ureter is dissected laterally from the broad ligament posterior leaf with the mesoureteric compartment, so the medial pararectal space is encountered, and the hypogastric nerve is identified. The obliterated umbilical artery is identified within the perivesical fatty tissue and dissected laterally so the medial paravesical space is developed. Developing the pararectal and paravesical spaces identify the lateral parametrium, the parauterine lymphovascular tissue with the uterine artery, the superior vesical artery, the superficial uterine vein, the paracervix with the deep uterine vein (or vaginal vein), the distal portion of the PSNs, and the proximal part of the IHP. The PSNs and the IHP are found dorsal to the deep uterine vein within the paracervix compartment.

The rectouterine pouch peritoneum is cut, and the rectovaginal septum is dissected. At this stage, the posterior leaf of the broad ligament is cut at the perirectal border. Therefore, the dorsal parametrium is identified between the medial pararectal space and the rectovaginal septum. The ventral portion of the dorsal parametrium is the rectouterine ligament, the fatty tissue without a suspensory function. This component should be cut in order to reach the dorsal portion of the dorsal parametrium, the rectovaginal ligament. Lateral to the rectouterine ligament, the mesoureter and the HN are identified, and lateral to the caudal portion of the rectovaginal ligament, the IHP is identified.

The vesicouterine pouch peritoneum is cut, and the vesicovaginal septum is dissected. The ventral parametrium is identified between the medial paravesical space and the vesicovaginal septum. The ventral portion of the ventral parametrium is the vesicouterine ligament, the fatty-lymphoid tissue containing the cervicovesical vessels which are in connection with the uterine artery, the superficial uterine vein, and the superior vesical vessels. The vesicouterine ligament can be clearly noticed after transection and craniomedial mobilization of the PALT and uterine artery. Once the vesicouterine ligament is cut and craniomedially mobilized, the distal ureter is noticed clearly, lying adjacent to the lateral margin of the upper vagina. Developing the paravaginal space between the lateral vaginal wall and the distal ureter is a key step to reaching the dorsal portion of the distal ureter, the VVL, the vesical branches of the IHP, the distal portion of the IHP, the paracolpium, and the entire paracervix. The VVL lies at the ventral aspect of the IHPvb, and the VVL may contain some parts of the paravaginal (vesicovaginal) veins, which drain into the vaginal vein (deep uterine vein). The vaginal vein lies within the paracervix. The paracolpium is the medial aspect of the paracervix, adjacent to the upper-middle vagina. Between the paracolpium and the VVL, the vesical branches of the IHP can be dissected; in this area, vesical nerve branches may lie within the network of vesical veins.

Pelvic parietal lymph nodes can be resected at the beginning of the procedure when the paravesical and pararectal spaces are developed. This facilitates the identification of parametrial limits with clear margins.

## 6. Complications

Complications of RH are divided into intraoperative and postoperative. Intraoperative complications include injury to the adjacent tissues such as the rectum, bladder, ureter, or the internal iliac vein and its tributaries. Iatrogenic damage to the internal iliac vein can happen during extensive resection of the LP (especially during paracervix resection) in Type C RH [70,71,72]. The tip of the surgical forceps could clamp the ventral wall of the internal iliac vein. Extensive bleeding could occur during resection of the clamped paracervix tissue, as the vessel is not visible. Therefore, it is advisable to perform initial pelvic lymph node dissection in order to better identify and mark the vascular system of the pelvis. Intraoperative injury to the bladder and rectum occurs during inappropriate dissection of the vesicouterine/vesicovaginal and rectovaginal spaces. The ureter should be identified and marked at the beginning of the procedure, as injury to the ureter may happen during almost every step of the procedure. However, an increased risk of ureteral injury is most commonly observed during the dissection of the ventral parametrium [18,31].

Postoperative complications include different types of fistulas (vesicovaginal, ureterovaginal, and rectovaginal), wound infection/dehiscence, urinary tract infections, postoperative ileus, and damage to the pelvic autonomic nerves [12,18,70]. The most common and specific postoperative complications of RH are ureterovaginal fistulas and damage to the PANS. Ureterovaginal fistulas result from devascularization of the ureter during its dissection up to the ureterovesical junction [12,25]. Additionally, using thermal energy (electric or ultrasonic energy devices) during the dissection of the ventral parametria is also associated with an increased risk of ureterovaginal fistulas. Iatrogenic damage to the PANS leads to postoperative urinary, anorectal, and sexual dysfunction (decreased vaginal lubrication and libido). Surgical steps in order to avoid injury to these nerves have been described above in detail. It is imperative to mention that dissection of the ventral parametria with scissors and sutures is associated with better postoperative function of the pelvic autonomic nerves (especially the vesical nerve branches) compared to dissection by using thermal energy devices [18].

RH is one of the most complex surgical procedures in pelvic surgery, with an increased incidence of perioperative complications compared to simple hysterectomy. Therefore, RH should be performed in high-volume gynecologic oncology hospitals with a particular focus on and training in gynecologic oncology surgery.

## 7. Conclusions

The Q–M classification of RH has provided a unique opportunity for uniform surgical and anatomical terminology. This classification offers detailed explanations of anatomical landmarks and resection margins for the three parametria of the uterus. Its worldwide acceptance could enhance surgical harmonization and precise standardization among oncogynecologists, which would facilitate accurate and comparable results of multi-institutional surgical clinical trials.

- The proposed update of the RH classification should reflect the resection of the PALT in Type A and B RH.
- Clarification of the anatomical landmarks between the paracervix and paracolpium is not easy; however, the paracolpium is the medial aspect of the paracervix adjacent to the upper-middle vagina.
- A precise anatomical description of the paracervical lymph nodes is needed, and a step-by-step guide for paracervical lymphadenectomy will help surgeons clearly understand its surgical application.
- The paracervical lymph nodes are located lateral/dorso-lateral from the axis of the internal iliac vessels.
- Paracervical lymphadenectomy should be an integral part of Type B and C RH and all RH types in which lymph node dissection is performed.
- A nerve-sparing approach can be adopted for Type C2 resection and should be included in the classification: Selective-Systematic Nerve-Sparing Type C2 RH, C2N. This could improve the radicality of parametrial/paracervical resection and decrease functional morbidity.
- Type D1 has no applications according to recent guidelines for CC treatment, but it still has a role in recurrent or persistent CC after definitive radiation or chemoradiation.
- Type D2 could be termed “modified LEER”—without resection of other organs and anatomical structures.

## Figures and Tables

**Table 1:** Different types of radical hysterectomy and resection lines of the three parametria between the Q–M classification and our update. PSNs—pelvic splanchnic nerves; HN—hypogastric nerve; LEP—laterally extended parametrectomy; LEER—laterally extended endopelvic resection.

**Types of RH According to Q-M Classification**	**Parauterine Tissue** **Paracervix**	**Vesicouterine/Vesicovaginal Ligaments**	**Rectouterine/Rectovaginal** **Ligaments**
Type A	Resection between the ureter and the pericervical adventitia. The ureter is not unroofed.	Vesicouterine ligament—minimal resection—5 mm Vesicovaginal ligament—not resected.	Rectouterine/Rectovaginal ligaments—minimal resection—5 mm.
Type B1	Resection at the ureteral tunnel. The ureter is unroofed.	Vesicouterine ligament—partial resection. Vesicovaginal ligament—not resected.	Rectouterine/Rectovaginal ligaments—partial resection.
Type B2	B1 plus paracervical lymphadenectomy.		
Type C1	At the level of the internal iliac vessels. At the level of the deep uterine vein.	Vesicouterine ligament—complete resection. Vesicovaginal ligament—only proximal (cranial resection).	Rectouterine/Rectovaginal ligaments—complete resection at the level of the rectum.
Type C2	The entire lateral parametrium is resected at the level of the internal iliac vessels.	Vesicouterine/Vesicovaginal ligaments—complete resection at the level of the bladder.	Rectouterine/Rectovaginal ligaments—complete resection at the level of the rectum.
Type D1—LEP	At the pelvic wall with transection of the internal iliac vessels.	Vesicouterine/Vesicovaginal ligaments—complete resection at the level of the bladder.	Rectouterine/Rectovaginal ligaments—complete resection at the level of the rectum.
Type D2—LEER	At the pelvic wall with transection of the internal iliac vessels. Resection of obturator fascia/muscle, coccygeus muscle, and sacrospinous ligament.	Vesicouterine/Vesicovaginal ligaments—complete resection at the level of the bladder.	Rectouterine/Rectovaginal ligaments—complete resection at the level of the rectum.
**Types of RH According to Our Update**	**Parauterine Tissue** **Paracervix**	**Vesicouterine/Vesicovaginal Ligaments**	**Rectouterine/Rectovaginal** **Ligaments**
Type A	PALT transected—if cannot be removed separately, the uterine artery and superficial uterine vein composing the parauterine tissue is removed together with the PALT at the level of the internal iliac artery Paracervix—resection between the ureter and the pericervical adventitia	Vesicouterine ligament—minimal resection—5 mm. Vesicovaginal ligament—not resected.	Rectouterine/Rectovaginal ligaments—minimal resection—5 mm.
Type B1	Parauterine tissue—at the level of the internal iliac artery in order to remove the PALT. Paracervix—at the level of the ureteral tunnel.	Vesicouterine ligament—partial resection. Vesicovaginal ligament—not resected.	Rectouterine/Rectovaginal ligaments—partial resection.
Type B2	B1 plus paracervical lymphadenectomy.		
Type C1	At the level of the internal iliac artery. At the level of the deep uterine vein. Paracervical lymphadenectomy PSNs and IHP are spared.	Vesicouterine ligament—complete resection. Vesicovaginal ligament—only proximal (cranial resection). Bladder nerve branches are spared.	Rectouterine/Rectovaginal ligaments—complete resection at the level of the rectum. The HN and IHP are spared.
Type C2	The entire lateral parametrium is resected at the level of the internal iliac artery. Paracervical lymphadenectomy PSNs and IHP are resected.	Vesicouterine/Vesicovaginal ligaments—complete resection at the level of the bladder. Bladder nerve branches are resected.	Rectouterine/Rectovaginal ligaments—complete resection at the level of the rectum. The HN is resected.
Type C2—selective-systematic nerve sparing	The entire lateral parametrium (parauterine/paracervix) is resected at the level of the internal iliac artery. Paracervical lymphadenectomy PSNs and IHP are selectively spared.	Vesicouterine/Vesicovaginal ligaments—complete resection at the level of the bladder. Bladder nerve branches are selectively spared.	Rectouterine/Rectovaginal ligaments—complete resection at the level of the rectum The HN is spared.
Type D—LEP or modified LEER	LEP—at the pelvic wall with transection of the internal iliac vessels. Modified LEER—at the pelvic wall with transection of the internal iliac vessels. Partial resection of the obturator fascia/muscle, and pelvic floor muscles—coccygeus muscle or sacrospinous ligament. No resection of other organs or anatomical structures (terminal ureter).	Vesicouterine/Vesicovaginal ligaments—complete resection at the level of the bladder.	Rectouterine/Rectovaginal ligaments—complete resection at the level of the rectum.

## Data Availability

The data presented in this study are available in this article.
